# Including gene networks to predict calving difficulty in Holstein, Brown Swiss and Jersey cattle

**DOI:** 10.1186/s12863-018-0606-y

**Published:** 2018-04-02

**Authors:** Francesco Tiezzi, Maria E. Arceo, John B. Cole, Christian Maltecca

**Affiliations:** 1 Department of Animal Science, North Carolina State University, Raleigh, NC 27695 USA; 2 Animal Genomics and Improvement Laboratory, ARS, USDA, Beltsville, MD 27705 USA

**Keywords:** Dystocia, Dairy cattle, Genome-wide association analysis, Biological networks

## Abstract

**Background:**

Calving difficulty or dystocia has a great economic impact in the US dairy industry. Reported risk factors associated with calving difficulty are feto-pelvic disproportion, gestation length and conformation. Different dairy cattle breeds have different incidence of calving difficulty, with Holstein having the highest dystocia rates and Jersey the lowest. Genomic selection becomes important especially for complex traits with low heritability, where the accuracy of conventional selection is lower. However, for complex traits where a large number of genes influence the phenotype, genome-wide association studies showed limitations. Biological networks could overcome some of these limitations and better capture the genetic architecture of complex traits. In this paper, we characterize Holstein, Brown Swiss and Jersey breed-specific dystocia networks and employ them in genomic predictions.

**Results:**

Marker association analysis identified single nucleotide polymorphisms explaining the largest average proportion of genetic variance on BTA18 in Holstein, BTA25 in Brown Swiss, and BTA15 in Jersey. Gene networks derived from the genome-wide association included 1272 genes in Holstein, 1454 genes in Brown Swiss, and 1455 genes in Jersey. Furthermore, 256 genes in Holstein network, 275 genes in the Brown Swiss network, and 253 genes in the Jersey network were within previously reported dystocia quantitative trait loci. The across-breed network included 80 genes, with 9 genes being within previously reported dystocia quantitative trait loci. The gene-gene interactions in this network differed in the different breeds. Gene ontology enrichment analysis of genes in the networks showed Regulation of ARF GTPase was very significant (FDR ≤ 0.0098) on Holstein. Neuron morphogenesis and differentiation was the term most enriched (FDR ≤ 0.0539) on the across-breed network. Genomic prediction models enriched with network-derived relationship matrices did not outperform regular GBLUP models.

**Conclusions:**

Regions identified in the genome were in the proximity of previously described quantitative trait loci that would most likely affect calving difficulty by altering the feto-pelvic proportion. Inclusion of identified networks did not increase prediction accuracy. The approach used in this paper could be extended to any instance with asymmetric distribution of phenotypes, for example, resistance to disease data.

**Electronic supplementary material:**

The online version of this article (10.1186/s12863-018-0606-y) contains supplementary material, which is available to authorized users.

## Background

Dystocia, or calving difficulty [1], has an important negative economic impact in the dairy industry. Births that require assistance result in increased veterinary costs, reduced longevity of cows, and increased mortality of calves [2, 3]. Difficult calving reduce the length of a cow’s productive life by 10% [2], mainly due to an increase in culling risk. Meyer et al. [4] and Bicalho et al. [5] found that dystocia had a major influence on stillbirth incidence of Holsteins, with the odds of a stillbirth being significantly greater than the odds of a live calf when the calf needed assistance. Holstein and Holstein crosses that experience difficult calvings also have increased median calving-conception intervals [5], number of services to conception, and times to first service [6]. All of these factors directly affect the profitability of a herd, mostly by reducing the lifespan of cows in the herd and by increasing the number of replacements needed.

Several contributing risk factors have been associated with dystocia [1, 7]. A disproportion in calf birth weight and maternal pelvic size is a major contributor to dystocia in domestic dairy cattle [1]. Calf birth weight is influenced by gestation length, which in turn, is influenced by paternal and maternal breed [1]. Both gestational length and dystocia have been shown to have a genetic component, and pedigree records have enabled the estimation of direct and maternal effects [8, 9]. In addition, genetic correlations have been identified between these functional traits and different conformation traits. Eaglen et al. [8] reported for example significant genetic correlations of gestation length with rump width, and of maternal calving difficulty with chest width, and with body depth. Furthermore, even assuming a similar heritability across breeds, there is an intrinsic variability for calving difficulty within breed. Different authors reported dystocia was highest for cows calving to Holstein bulls while Jersey calves caused the least dystocia [10–12]. As a result a search for quantitative trait loci (QTL) associated with dystocia in dairy cattle produced results only in Holsteins [9]. Indeed, at least 27 QTL were reported across the genome in Holstein-Friesian cattle, but to the best of our knowledge, none in Brown Swiss or Jersey.

Genome-wide association studies (GWAS) have been a valuable tool to detect single nucleotide polymorphisms (SNP) associated with specific phenotypes. Key functional mutations may be revealed by GWAS, assuming a sufficiently large number of individuals and dense SNP panels were available. However, for complex traits where a large number of genes are expected to influence the phenotype each with a small absolute effect, GWAS studies showed to be limited [13]. Genomic information has been officially incorporated into genetic evaluations of US Holstein, Brown Swiss and Jersey cattle since 2009 [14], and Ayrshires since 2013 [15]. The success of genomic selection relies on the accuracy of the SNP effects calculated and the linkage disequilibrium (LD) between the SNP and the QTL for the trait [16]. Patterns of LD vary among breeds, especially when these breeds have undergone divergent selection for many generations and the effective population size is small [17]. Genomic selection becomes important especially for complex traits of low heritability, such as fertility and health traits, where the accuracy of conventional selection is lower than for production traits [18, 19]. In addition, using multi-breed reference populations [20, 21] has been proposed as a way to ensure robust predictions when the reference population is small in size.

An appealing approach to enhance genomic predictions is to improve prediction using insight of the underlying molecular mechanisms of complex traits [22]. Biological networks in principle should better capture the genetic architecture of complex traits, being less dependent on LD patterns characteristics of a population. In this regard, correlation networks have been widely used both with gene expression [23, 24] or genotype data to integrate information from different levels. Fortes et al. [25, 26] used genotype data in cattle to unravel biological networks related with fertility traits and puberty. Constructing relevant within and across-breed gene networks for these three breeds related to dystocia may provide insight into the biology of the trait and produce robust predictions for dystocia in different cattle breeds. The objective of this study is therefore to identify and characterize breed specific and across-breed gene networks influencing dystocia in Holstein, Brown Swiss and Jersey cattle and verify if their inclusion in genomic prediction increase accuracy of prediction.

## Methods

A schematic representation of the overall set of analyses is provided in Additional file 1: Figure S1.

### Animals and phenotypes

Pseudo-phenotypes used in this project were derived from national genetic evaluations of calving difficulty, gestation length and conformational traits for three common breeds of US dairy cattle: Holstein (HO), Brown Swiss (BS) and Jersey (JE). Six traits previously associated with dystocia were employed, namely: calving difficulty (direct sire -DCD- and maternal grand sire -MCD-), gestation length (GL), and conformation traits (stature -STAT-, strength -STR- and rump width -RW-). The genetic merit of selected bulls was reported as predicted transmitted ability (PTA) and was provided by the Animal Genomics and Improvement Laboratory (AGIL), USDA. A detailed description of the different traits in selected bulls can be found at https://www.uscdcb.com/what-we-do/genetic-evaluations/. Briefly, calving difficulty was recorded on a scale of 1 to 5 (the larger the score the more difficult the calving) and a sire-maternal grand sire threshold model was fitted to the data to obtain the PTA [27]. The PTA was reported as the percentage of births that are difficult (calving difficulty score ≥ 4) for first-calf heifers. The model implemented allowed the estimation of both, sire and a maternal grand sire effect. This resulted in the availability of two pseudo-phenotypes for calving difficulty, DCD and MCD, respectively for the direct and maternal component. Gestation length (GL) is the direct effect on the interval from conception to parturition and it was derived from breeding and calving data [28]. Conformational (type) traits considered were stature (STAT), strength (STR) and rump width (RW). For type traits, the PTA was calculated with a multi-trait animal model [29, 30]. The PTA was a function of each bull’s daughters’ deviations from the base mean and of his parent’s average.

### De-regression

The PTAs for all traits were transformed into de-regressed PTA (dPTA), removing the parent average effect from contribution to the PTA [31] as well as the PTA’s variance shrinkage. The de-regression procedure produced weights (*w*) obtained using a *c* parameter of 0.5 [31]. The *c* parameter reflects the predictive ability of the genetic covariates, a large *c* results in overemphasis of less accurate information whereas a small *c* results in too little emphasis on less accurate results [31, 32]. When the parent average (PA) was not available, it was estimated as $\widehat{PA}$ = (0.5*sire PTA) + (0.25*maternal grandsire -MGS- PTA) + (0.25*average year of birth -AYB- PTA) [18], $\widehat{PA}$ = (0.5*sire PTA) + (0.5*AYB PTA), or $\widehat{PA}$ = (0.25*MGS PTA) + (0.75*AYB PTA) depending on data availability. Only records with a reliability of the de-regressed PTA larger than 0.2 and only animals with pseudo-phenotypes in all traits (to avoid an animal-trait confounding effect) were kept for the analyses. After editing, there were 8780 HO bulls, 505 BS bulls, and 1818 JE bulls with pseudo-phenotypes available. Pseudo-phenotypic data summary statistics are shown in Table 1. Correlation among traits is shown in Additional file 2: Figure S2.

**Table 1:** Mean and standard deviation of de-regressed PTA and de-regressed reliabilities for the phenotypes used ($\overline{x}$ ± *sd*)

	Holstein		Brown Swiss		Jersey	
Traits^a^	PTA	REL	PTA	REL	PTA	REL
DCD	7.95 ± 2.40	0.76 ± 0.13	5.32 ± 2.03	0.62 ± 0.17	4.94 ± 1.11	0.39 ± 0.17
MCD	8.02 ± 2.70	0.64 ± 0.15	5.34 ± 1.96	0.56 ± 0.18	5.16 ± 1.99	0.40 ± 0.17
GL	0.57 ± 0.58	0.93 ± 0.13	0.44 ± 0.83	0.74 ± 0.21	0.50 ± 0.55	0.86 ± 0.15
STAT	0.34 ± 1.64	0.85 ± 0.10	0.02 ± 1.67	0.83 ± 0.13	−0.06 ± 1.59	0.85 ± 0.11
STR	0.17 ± 1.47	0.83 ± 0.11	0.04 ± 1.12	0.73 ± 0.18	0.05 ± 1.06	0.80 ± 0.14
RW	0.22 ± 1.58	0.83 ± 0.10	−0.09 ± 1.01	0.70 ± 0.18	0.02 ± 0.99	0.78 ± 0.14
Number of Bulls	8780		505		1818	

^a^Traits included in this study were: direct sire calving difficulty (DCD), maternal grand sire calving difficulty (MCD), gestation length (GL), stature (STAT), strength (STR), and rump width (RW)

### Genotypes and genome-wide association analysis

Single nucleotide polymorphism (SNP) genotypes from different chips [14] were provided by the USDA’s AGIL. A total of 35,565, 34,665 and 41,580 quality-controlled SNP genotypes were available for BS, JE and HO bulls, respectively. Quality control included removing SNPs with minor allele frequency lower than 5% and call rate lower than 90%.

The genome-wide association was done on a single-trait basis within each of the three breeds. We used a Bayes-B implementation in GenSel software [33] such that,

$$ \mathrm{y}={\mathbf{1}}_{\mathbf{n}}\upmu +\sum \limits_{\mathrm{i}=1}^{45188}{\mathrm{Z}}_{\mathrm{i}}{\mathrm{g}}_{\mathrm{i}}+\mathbf{e} $$

where **y** is a vector of dPTAs, *n* is the number of bulls (8,780,505, or 1818), μ is the overall mean, **g** is a vector with random SNP effects with their variance conditional on π = 0.9, **Z** the incidence matrix of genotypes and **e** is a vector of residuals, considered to be normally distributed *N*(**0**, **R**σ_e_^2^) with R_ii_ = 1/*w*_*i*_ and σ_e_^2^ ~ *X*^− 2^(v = 10, *S*). The conditional marker variance at each iteration was

$$ {\sigma}_g^2\pi \sim \left(\begin{array}{cc}0& p\left(\pi \right)\\ {}{vS}^2{X}_v^{-2}& p\left(1-\pi \right)\end{array}\right) $$

in which *X*^*− 2*^ is an inverted chi-squared distribution with v = 4 and *S* = [σ^2^ *(v – 2)]/*v* with σ^2^ = 5 for σ_g_^2^ and σ^2^ = 3 for σ_e_^2^.

A total of 40,000 samples were obtained from the Gibbs sampler, 5000 of which were discarded as burn-in. We did not expect the results to be very sensitive to the chain length, due to the relatively high reliability of the pseudo-phenotypes. However, a preliminary analysis was done with 50,000 iterations and 10,000 as burn-in for 3 traits per breed and results did not change (results not shown). A proxy for the proportion of genetic variance (PV_g_) accounted for by each SNP was calculated as:

$$ {\mathrm{PV}}_{\mathrm{g}}=\frac{\left[2\mathrm{p}\left(1-\mathrm{p}\right)\right]{\overline{\mathrm{a}}}^2}{\sum_1^{45188}\left[2\mathrm{p}\left(1-\mathrm{p}\right)\right]{\overline{\mathrm{a}}}^2} $$

where $\overline{a}$ = posterior mean of the effect for that locus averaged across the post burn-in samples and p is the major allele frequency.

For each breed/trait a lenient significance threshold was arbitrarily set at the 75th percentile of the sample, that is, we retained the top 25% of SNP (corresponding to a count of 11,297 SNP) for further analyses.

### Mapping of SNP to genes

Each SNP in the chip was mapped to its closest annotated gene using the *Bos taurus* UMD3.1 assembly [34] (http://www.ensembl.org/index.html). Criterion for mapping consisted in a 5′ or 3′ maximum distance to the nearest gene smaller or equal to 2500 bp. A total of 15,634 SNP were mapped to a total of 8599 genes. In the case of SNP mapped to more than one gene, all genes selected were retained. In the case of genes mapped by more than one SNP, a SNP filter was applied to preserve the “one SNP to one gene” rule (see below).

### Association weight matrix and gene networks

In general, the construction of an association weight matrix (AWM) followed the procedures previously implemented by Fortes et al. [26]. For each of the three breeds, the construction of the AWM started with the selection of relevant SNP from those identified as significant in the association analyses. Relevant SNP selected were the ones which met at least one of the three following criteria: a) were significant for all traits evaluated, b) were significant for all of the functional traits evaluated (DCD, MCD, and GL), c) were significant for all the type traits evaluated. Each column in the AWM corresponded to a trait. Each row corresponded to a SNP. Each cell in the matrix corresponded to the *z*-score normalized effect size for the particular SNP. The effect size was defined as the posterior mean of the SNP effect averaged across the post burn-in chains. Then, each row in the AWM was indexed with its gene. When different relevant SNP were mapped to the same gene, the most significant relevant SNP was assigned to that gene (“one SNP to one gene” rule). The most significant relevant SNP of a particular gene was defined as the one with the largest PV_g_ on average across all traits. When relevant SNP were not mapped to a gene but they met criterion a), they were kept in the AWM. Therefore, the AWM_m*x*t_ had as many rows as [indexed genes + SNP meeting criterion a)] and as many columns as traits (6). Subsequently, row-wise partial correlations were computed on the AWM matrix using the PCIT algorithm [35] in R [36]. This algorithm allowed identification of significant partial correlations and produced an *m* symmetric adjacency matrix. Each cell in the adjacency matrix corresponded to a partial correlation between gene *i* and gene *j*. When partial correlations were not significant the value in the cell was set to 0. The significant correlations identified could be interpreted as significant gene-gene interactions (connections). These interactions put together resulted in a gene network.

Finally and since with the PCIT algorithm better sensitivity is achieved with larger number of traits [35], to avoid spurious connections we trimmed the network to a sub-network consisting only of connections with a partial correlation (r_xy|z_) equal or larger than 0.98. We computed 4 networks, 1 per breed (breed-specific networks) and 1 for the intersection of the 3 breeds, that is, genes shared among the 3 breed-specific networks (across-breed network). To ensure that genes in the across-breed network were not a product of chance we tested the intersection size using a variant of the Binomial distribution [37]. This statistic models the intersection sizes when sampling from different sets without replacement. For visualization of results we used R-igraph [38].

### Gene ontology (GO) enrichment analyses

For each breed, we used the genes in the adjacency matrix to search for enriched functional annotation. Human homologs of these genes were retrieved, and used to evaluate enrichment of the GO biological process branch using GOstats [39]. To account for the false positives resulting from the multiple testing, we used a false discovery rate (FDR) [40] of ≤35% in Holstein and Jersey. We used FDR ≤ 60% in Brown Swiss due to the smaller power of the analysis resulting from a smaller *n* when compared to the other breeds. Human homologs were used due to the richer annotation as compared to cattle. The human homologs were contrasted to a background list containing all genes from the *Homo sapiens* GRCh38 assembly representing the 8599 *Bos Taurus* genes. To reduce dimensionality and redundancy in the lists of enriched GO terms we used REVIGO [41] with a C value of 0.5 (C is a user specified proportion of terms remaining in the list after the algorithm has finished the cluster representatives. A lower (more stringent) value of C will result in a shorter, but also a more semantically diverse list [41]) and the sim_Rel_ measure of semantic similarity [42]. Semantic similarity measures exploit GO structure to compare concepts within an ontology based on common ancestors, to reduce functional redundancies. The terms are assigned to clusters based on a semantic similarity measure. At C = 0.53 there is 99% chance an above-background similarity exist between each pair of terms in a cluster [41]. The semantic similarity threshold at which the terms were removed from a list and assign to a cluster corresponds to the dispensability. Cluster representatives always have dispensability less than C [41]. Therefore, the smaller the dispensability the more representatives were the terms.

### Mapping of genes to dystocia QTL

For each breed, we retrieved the genes for enriched GO terms and evaluated their location in the genome. Specifically, we searched for genes located within dystocia QTL for the UMD3.1 assembly available at the CattleQTLdb [9] (http://www.animalgenome.org).

### Pathway analysis

For all 4 networks we evaluated biological pathway enrichment (FDR ≤ 0.35). We used the genes in each breed-specific network that were within dystocia QTL and all other genes interacting with them. In addition, we evaluated pathway enrichment by the 80 genes in the across-breed network. We used a combined measure that evaluated KEGG pathway overrepresentation as well as KEGG pathway perturbation to assess significance [43]. In the breed-specific networks, the measure of change (ΔEg_i_) used to compute the perturbation in gene *i* was the average allelic effect across traits. In the across-breed network, ΔEg_i_ corresponded to the average allelic effect across breeds and traits. We evaluated enrichment in two species with rich annotation, human and mouse.

### Gene networks in genomic predictions

Genomic prediction of bulls’ genetic potential was performed with the inclusion of network information in order to investigate any potential increase in accuracy deriving from including network information through a GBLUP model.

The models were tested using a 4-fold cross-validation scheme. For each breed, bulls were separated into four medoids (families) by k-means based on the pedigree-based relationship matrix [44], thus creating four training-validation sets within each breed. Number of bulls masked in each validation set was 122, 177, 88, 118 for BS, 317, 633, 496, 371 for JE and 2426, 2083, 1986, 2285 for HO.

The genome-wide association analysis and association weight matrix construction was repeated for each training set, and served to construct models for the prediction of PTA in the validation set. Different models were tested. A general genomic relationship matrix **G** [45] was used as reference method and built on the whole set of markers (BASE). Markers were then gathered if ranking among the top 25% for PV_g_ for at least three of the six traits considered, a genomic relationship matrix was built on these markers (TOP25) as well as on the remaining markers (BOT75) [32]. Network information was first included by restricting the set of markers to those mapped a gene and retained after the construction of the AWM. The genomic relationship matrix was constructed by including only these markers (NET) or all the remaining markers (FREE). Matrices (BASE, TOP25, BOT75, NET) were constructed using the first method reported by VanRaden [45]. The second approach (CONN) included AWM correlations among markers (genes) in building the **G** matrix, in order to represent genes that were connected in the network and increase their contribution to the overall genomic variation. This matrix was built following:

$$ \boldsymbol{G}={\boldsymbol{ZDZ}}^{\hbox{'}} $$

being **Z** a matrix of centered and scaled genotypes [45] for the set of markers in the network and **D** the AWM, with ‘1’ values on diagonal and pairwise gene correlations on the off-diagonals.

In order to provide same scale as the other matrices, CONN was scaled using the allele frequency-free method described by VanRaden [45] using the BASE matrix as reference.

Models were built including different combinations of effects (matrices) as reported in Table 2. Briefly, each model included one (models 1, 2, 4 and 6) or two (models 3, 5 and 7) animal additive genetic effects, that differed in the genomic relationship matrix used. Variance components for each effect within model-trait-training set combination were estimated using Gibbs Sampler as implemented in R package BGLR (Pérez and de los Campos, 2014). Model predictor was calculated as the sum of the estimates of the intercept and the genetic effect(s) as included in each model. Prediction was assessed by masking dPTA in training set in a 4-fold cross-validation, accuracy of prediction was measured as the correlation between the predicted and masked real dPTA.

**Table 2:** Genomic relationship matrix (G) calculated and employed in the prediction of calving difficulty

Model/G	BASE	TOP25	BOT75	NET	CONN	FREE
Model-1	X	.	.	.	.	.
Model-2	.	X	.	.	.	.
Model-3	.	X	X	.	.	.
Model-4	.	.	.	X	.	.
Model-5	.	.	.	X	.	X
Model-6	.	.	.	.	X	.
Model-7	.	.	.	.	X	X

BASE was a general genomic relationship matrix was used as reference method and built on the whole set of markers. Markers were then gathered if ranking among the top 25% for proportion of genetic variance explained for at least three of the six traits considered, a genomic relationship matrix was built on these markers (TOP25) as well as on the remaining markers (BOT75). A genomic relationship matrix was constructed by including only the markers that were in proximity of a gene and therefore included in the network (NET) or all the remaining markers (FREE). Matrix CONN included NET markers also accounting for AWM correlations among markers (genes)

## Results

### Genome-wide association analysis

Significant SNP showed an average PV_g_ across traits larger or equal than 4.54 *×* 10^− 6^ in HO, 1.79 *×* 10^− 5^ in BS, and 1.18 *×* 10^− 5^ in JE. The distribution of PV_g_ averaged across all traits for each SNP is shown in Additional file 2: Figure S2. In each breed, few SNP showed an average PV_g_ across traits comparatively larger than the rest (Table 3). Manhattan plots for all traits in all breeds are reported in Additional file 3: Figure S3, Additional file 4: Figure S4, Additional file 5: Figure S5, Additional file 6: Figure S6, Additional file 7: Figure S7, Additional file 8: Figure S8, Additional file 9: Figure S9 and Additional file 10: Figure S10. In HO, the SNP that explained the greatest average PV_g_ across traits was on BTA18 at 57.5 Mbp. This SNP is located within three previously described QTL strongly associated with calving difficulty [46–49]; and close to QTL for gestation length [50], birth index [47, 48], birth weight [51] and conformation [46]. Furthermore, when looking within a genome window of size 8 Mbp centered on 57Mbp (53–61 Mbp), our results were consistent with another QTL described to be affecting calving difficulty in European Holstein cattle [52] and more specifically to QTL related to dystocia [46, 53, 54]. Likewise, SNP in BTA5 explained a large amount of PV_g_ in HO and JE. In both breeds the significant SNP were located around 106 Mbp. A QTL affecting calving difficulty and related to traits affecting body size was reported to be located ~ 70.8 Mbp on BTA5 for Holstein Friesian [55]. In addition, a genomic region was described at ~ 140 Mbp to be associated with calving interval in a HO-JE GWAS [56]. In BS the 4 most significant SNPs were located on BTA17 at 68.1and 68.2 Mbp, and in BTA25 at 1.4and 1.6 Mbp. Genomic regions were described in Holstein-Friesian associated with dystocia in BTA17 at 2.7 and 37 Mbp. A QTL associated with dystocia was also described in Holstein BTA25 at 6.3 Mbp [53]. No QTL associated with reproductive performance or conformation were reported in BS in BTA17 in the neighborhood of our significant SNPs. However, a strong signal has been found on BTA25 of BS, for QTL associated with production and conformation traits [57]. Specifically, a QTL associated with body depth spans the region between 1.1 and 2.9 Mbp where our most significant SNP were located. In JE, no related QTL were described in BTA15, BTA9, or BTA4. A QTL associated with gestation length in BTA5 spanning 26.4–26.6 Mbp was described in a Jersey-Limousin cross [58].

**Table 3:** Top 5 SNP explaining the larger proportion of genetic variance averaged across traits in Holstein (HO), Brown Swiss (BS), and Jersey (JE)

Breed^a^	SNP rs	Mapped to gene (gene name)	Chr^a^	Location (bp)	Average PV_g_^b^
HO	rs109478645	ENSBTAG00000037537	18	57589121	0.148
	rs109293774		5	106178425	0.014
	rs41257416	ENSBTAG00000005465 (*NDUFA9*)	5	105870613	0.011
	rs42196507		29	50296573	0.010
	rs111027600		6	109682953	0.010
BS	rs110914965	ENSBTAG00000020619 (*LOC504986*)	25	1665327	0.026
	rs109557202	ENSBTAG00000019249 (*HS3ST6*)	25	1489008	0.010
	rs109165051		17	68143571	0.005
	rs109646366		17	68221835	0.004
	rs43709092		4	12868626	0.003
JE	rs43032701	ENSBTAG00000011873 (*KCNE3*)	15	54589414	0.075
	rs41621381		4	2132412	0.058
	rs110421124	ENSBTAG00000016649 (*CCND2*)	5	106269362	0.010
	rs110702021		5	106296860	0.007
	rs109857460	ENSBTAG00000015242 (*SLC18B1*)	9	71939142	0.005

^a^Chr: chromosome

^b^PV_g_ is the proportion to 1

### Gene networks

**Fig. 1:**
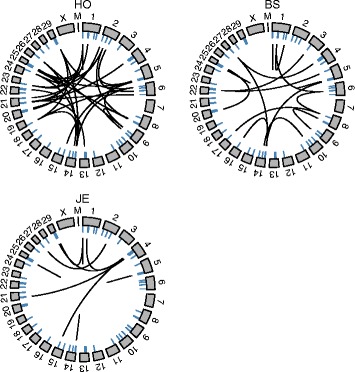
Circular plots of the cattle genome, showing length of chromosomes (grey rectangles), location of genes on the across-breed network (blue lines), and interactions among genes in Holstein (HO), Brown Swiss (BS), and JE (Jersey)

**Fig. 2:**
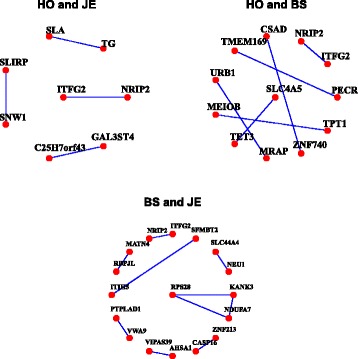
Genes and gene-gene interactions shared by all possible breed pair combinations

For complex traits like dystocia, a large number of genes are expected to influence the phenotype, each with a small absolute effect. Therefore, GWA studies would only partially unravel molecular features affecting the trait [13]. Biological networks may better capture the genetic architecture of complex traits. Breed-specific dystocia gene networks included 1272 genes in HO, 1454 in BS and 1455 in JE. Their number of connections (the degree of the vertices induced by the PCIT algorithm) ranged between 1 and 80 in HO, 1 and 17 in BS, 1 and 13 in JE. Biological networks usually follow a Power-law distribution [59]. Although it is in general hard to see that a distribution is a Power-law when networks are not big enough, we used a Kolmogorov-Smirnov test to validate this assumption, and the null hypothesis of the networks being drawn from a Power-law distribution was not rejected [38]. Of this larger number of genes total of 256 genes in HO, 275 in BS, and 253 in JE were associated with reported dystocia QTL (regardless of breed). Furthermore, 9 of these genes in HO, 2 in BS, and 3 in JE were among the most-connected ones in the network -top 5% of the sample for HO and JE, top 10% for BS- (Table 4). None of the top 5 SNP explaining the larger average PV_g_ in the GWAS were among the most connected genes/SNP in the networks. The HO network had 2 well-defined clusters of highly connected genes, whereas clustering in the BS and JE networks seemed less defined. Clusters included many genes that were enriching significant GO terms as well as genes located within dystocia QTL (data not shown). However, we should point out that the different number of bulls from each breed used in these analyses may have impacted the structure of the networks, such that in the HO with a significant larger sample size, more interactions could be detected. In addition to constructing breed-specific networks, we explored an across-breed dystocia network consisting of genes shared among the breed-specific networks. There were 80 genes at the intersection of the three networks. The probability of an intersection of size 80, given the size of the breed-specific networks, was highly significant (*p* ≤ 7.9e-11) making it a very unlikely event to occur by chance [37]. For these 80 genes, the gene-gene interactions differed among breeds. That is, interactions among genes inside the intersection and interactions among genes within the intersection with other genes outside of it varied in the three breeds (Fig. 1). A total of 9 genes in the intersection were within reported dystocia QTL (Table 5). Additionally, a more stringent across-breed network would include only the sub-network of these genes that are connected in the same way in the 3 breeds. In our study no such sub-network was identified. However, when looking at 2 breeds at a time, we found that small sub-networks of between 7 and 17 vertices existed for all three possible pair-wise combinations (Fig. 2).

**Table 4:** Most-connected (top 5% of the sample for HO and JE, top 10% for BS) genes in the breed-specific networks, located within dystocia QTL

Breed	Ensembl ID	Gene name	Degree	Chr^a^	Gene location in bp (start-end)	QTL ID
Jersey	ENSBTAG00000027654	*EIF4EBP1*	12	27	32951594–32973435	11393
	ENSBTAG00000007602	*ITGA8*	7	13	30340780–30527982	11385
	ENSBTAG00000009987	*ITGB3*	7	19	46968837–47027388	11367
Holstein	ENSBTAG00000018863	*PRIMPOL*	78	27	14110851–14163989	11393
	ENSBTAG00000009565	*RASA1*	77	7	89281002–89391377	2699
	ENSBTAG00000031632	*ADRA1A*	72	8	75257161–75364423	11442
	ENSBTAG00000021237	*DST*	71	23	3431407–3687495	11390
	ENSBTAG00000021699	*RORB*	66	8	50875138–5107931	11442
	ENSBTAG00000018669	*PLB1*	65	11	71116627–71221929	11384
	ENSBTAG00000012005	*IFT43*	63	10	88272302–88379899	11444
	ENSBTAG00000021811	*ANGPT4*	62	13	60729756–60773778	11385
	ENSBTAG00000013079	*CTCFL*	55	13	59193465–59217987	11385
Brown Swiss	ENSBTAG00000019044	*BAIAP2*	11	19	52198618–52263294	11368
	ENSBTAG00000009960	*FLOT1*	9	23	28090029–28100113	11390

^a^Chr: chromosome

**Table 5:** Genes in the cross-breed network located within dystocia QTL

Ensembl ID	Gene name	Chr^a^	Gene location in bp (start-end)	QTL ID
ENSBTAG00000040543		9	69513253–69514728	11352
ENSBTAG00000025403	*TTLL5*	10	87923923–88238514	2702
ENSBTAG00000011106	*PACRG*	9	99649262–100180707	11352
ENSBTAG00000020829	*PTPRK*	9	66968686–67594209	11352
ENSBTAG00000027444	*SVIL*	13	34860211–34965892	11385
ENSBTAG00000007013	*TOM1L1*	19	5221507–5283308	11368
ENSBTAG00000007937	*PRIM2*	23	2475807–280986	11390
ENSBTAG00000010241	*UNC5D*	27	30421577–31075903	11393
ENSBTAG00000021237	*DST*	23	3431407–3687495	11390

^a^Chr: chromosome

### GO and pathway enrichment analyses

We used the genes within the breed-specific and across-breed networks to search for enriched GO terms (FDR ≤ 0.35 HO and JE; 0.6 BS). In this analyses we used a relatively high FDR to account for sample size disparities among breeds and still allow the interpretation of the data in the context of biological processes. A total of 162 GO biological process branch terms were enriched in HO, 22 in BS, and 33 in JE. Enriched GO terms comprised 856 in the HO network, 133 in the BS network, and 490 in the JE network. A total of 185 of these genes were located within 24 reported dystocia QTL in HO, 31 were within 14 reported dystocia QTL in BS, and 86 were within 18 reported dystocia QTL in JE. In the HO breed 2 terms stood out as significant: regulation of ARF GTPase activity (FDR = 0.0098) and regulation of ARF protein signal transduction (FDR = 0.0584). Similarly, in the BS regulation of ARF GTPase activity was among the most significant for the BS dystocia network. ARF GTPases are within the Ras superfamily of GTP-binding proteins. ARF proteins localize to membranes in the cells and regulate the actin cytoskeleton and vesicle trafficking [60–62]. The actin cytoskeleton plays an important role in the formation of the immunological synapsis [63]. The immunological synapsis is key in the recognition of antigens and the articulation of an effective immune response, ARF GTPase regulators have been previously reported to be involved in vesicular secretion regulation of T cells [63]. In the JE, no terms were significant. The genes in the JE network were mainly enriched in terms related to muscle and nervous system development, such as striated muscle cell development, regulation of calcium ion-dependent exocytosis, neuron projection morphogenesis, and microtubule bundle formation. The source of this signal was most likely the skeletal muscle. Differences in the prenatal fiber development among breeds of dairy and beef cattle have been described [64]. In addition, differences in fibrogenesis were also described in Wagyu versus Angus cattle [65]. Most research focusing on skeletal muscle has mostly involved beef cattle, but differences in skeletal muscle development of Jerseys compared to other dairy breeds may be possible. Furthermore, this signal may be targeting differences between HO-BS and JE during the active stage of labor when voluntary abdominal straining starts. Genes in the across-breed network enriched 370 GO terms (FDR ≤ 0.35). The most significantly enriched terms were related to neuron morphogenesis and differentiation (FDR ≤ 0.0539). We then reduced these lists of GO terms to 46 non-redundant terms in HO, 7 in BS, 10 in JE and 93 in the across-breed. The most significant non-redundant terms (cluster representatives) in each breed are presented in Table 6.

**Table 6:** Top non-redundant terms for the GO biological process branch. For Holstein, Jersey and the cross-breed networks, the terms correspond to a FDR ≤ 35% and a dispensability = 0. For Brown Swiss, the terms correspond to a FDR ≤ 60% and a dispensability = 0

Network	Description	GO term id	*P*-value
Jersey	Striated muscle cell development	GO:0055002	0.0004
Brown Swiss	Regulation of ARF GTPase activity	GO:0032312	0.0003
	Establishment of vesicle localization	GO:0051650	0.0005
Holstein	Regulation of ARF GTPase activity	GO:0032312	< 0.00001
	Cell adhesion	GO:0007155	0.0061
	Biological adhesion	GO:0022610	0.0061
Aross-breed	Cell morphogenesis involved in neuron differentiation	GO:0048667	0.0001
	Negative chemotaxis	GO:0050919	0.0070
	Protein localization to cell surface	GO:0034394	0.0132
	Locomotion	GO:0040011	0.0340
	Developmental process	GO:0032502	0.0492
	Cell-cell adhesion	GO:0098609	0.0495
	Localization	GO:0051179	0.0860

Regarding pathway enrichment, in the HO and JE networks several pathways were overrepresented, whereas in the BS network none was overrepresented at the significance threshold. Most pathways overrepresented were shared in mouse and human (Table 7). In HO, the most significant pathway was cell cycle (FDR ≤ 0.002). In the across-breed network, overrepresented pathway was Shigellosis. Although other pathways in this network did not make the significance threshold, top pathways showed a consistent trend towards resistance to infection as being involved in molecular differences underlying dystocia in dairy cattle. Top pathways in the across-breed network included: Bacterial invasion of epithelial cells (hsa05100), Regulation of autophagy (hsa04140), Gap junction (hsa04540) and Regulation of actin cytoskeleton (hsa04810).

**Table 7:** Top KEGG pathways (FDR ≤ 0.35). When pathways were enriched in both species, mouse and human, we listed the one with the smaller FDR

Network	Pathway	KEGG ID	FDR
Holstein	Cholinergic synapse	hsa04725	0.188
	Cell cycle	mmu04110	0.002
	Morphine addiction	hsa05032	0.249
Jersey	Amoebiasis	hsa05146	0.185
	Small cell lung cancer	mmu05222	0.001
	ECM-receptor interaction	hsa04512	0.185
Across-breed	Shigellosis	hsa05131	0.318

Our GWAS results showed that SNP explaining the largest proportion of genetic variance were also within or in the proximity of reported QTL directly or indirectly associated with size. That is QTL for calving difficulty, birth index, body size, and body depth. All these QTL would potentially add to a feto-pelvic disproportion. And yet, it has been shown that there are some Holstein bulls that could produce the same (lower) incidences of dystocia in their progeny as some Jersey bulls [12]. Results from the biological networks analysis pointed to a link among dystocia, bacterial infection and alteration of muscle functionality. That is, sires might not only influence the feto-pelvic proportion but may also be influencing the response of their female progeny to certain environmental factors.

### Variance absorbed by gene networks

Variance absorbed by the different genomic relationship matrices in the cross-validation scheme is reported in Additional file 11: Table S1, Additional file 12: Table S2 and Additional file 13: Tables S3 for HO, JE and BS, respectively. The value reported in the average value (SD) of the posterior means as repeated by excluding each of the 4 validation sets subsequently.

Variance explained by the genomic effect was generally higher for type traits (from .57 to .87 across trait/breeds/model when employing the BASE **G**), intermediate for calving difficulty traits (from .08 to .43 across trait/breeds/model when employing the BASE **G**) and low for GL (from .06 to .14 across trait/breeds/model when employing the BASE **G**). Estimates on HO showed smaller variation across the replicates, whereas this variation was intermediate for JE and higher for BS, probably due to reduced sample size. Trend in variance absorbed by the different effects was moderately consistent across breeds. Effects TOP25, NET and CONN were the effects absorbing the highest proportion of variance, BASE was intermediate and BOT25 and FREE were absorbing the lowest. Some models included two different effects to assess complementarity between the 2 sets of markers. In that case, effects TOP25, NET and CONN generally absorbed more variance than BOT75 and FREE.

### Gene networks in genomic prediction

Prediction accuracy is reported in Table 8. Pattern across models and traits were consistent, with HO showing the highest accuracies among the three breeds. Prediction accuracy was higher for type traits, lower for GL and intermediate for calving difficulty traits. Prediction accuracy did not increase or decrease significantly across models. A slight decrease was observed when including network information (NET and CONN), which was recovered when complementary effect was included (FREE). An attempt to include the across breed network in genomic prediction resulted in no increase in accuracy and a slight decrease for the Holstein breed (results not shown). While there is relatively sizable body of evidence in the use of AWM to identify potential candidate genes and pathways [66, 67], their use in genomic prediction is somewhat more limited. Snelling and colleagues [68] found that including AWM and gene networks resulted in an increase in prediction accuracies of approximately 10–15% according to trait for beef tenderness traits. Related but simplified approaches based on marker effect SNP-weighting for production traits in US Holstein have often obtained marginal increases in accuracies [32].

**Table 8:** Prediction accuracy (SE) for the different combination of traits network genomic relationship matrix for Holstein, Jersey and Brown Swiss

	Model-1	Model-2	Model-3	Model-4	Model-5	Model-6	Model-7
Holstein							
DCD	0.47 (0.03)	0.46 (0.03)	0.46 (0.03)	0.43 (0.02)	0.46 (0.02)	0.34 (0.02)	0.44 (0.03)
MCD	0.54 (0.03)	0.52 (0.03)	0.52 (0.03)	0.48 (0.03)	0.53 (0.03)	0.38 (0.03)	0.52 (0.02)
GL	0.21 (0.08)	0.2 (0.07)	0.21 (0.07)	0.19 (0.06)	0.21 (0.07)	0.13 (0.03)	0.18 (0.05)
STAT	0.76 (0.02)	0.73 (0.02)	0.73 (0.02)	0.68 (0.02)	0.75 (0.02)	0.57 (0.03)	0.74 (0.02)
STRENGTH	0.66 (0.02)	0.63 (0.02)	0.64 (0.02)	0.57 (0.02)	0.65 (0.02)	0.48 (0.03)	0.63 (0.02)
RUMPWIDTH	0.7 (0.02)	0.67 (0.02)	0.68 (0.02)	0.62 (0.03)	0.69 (0.02)	0.52 (0.02)	0.68 (0.02)
Jersey							
DCD	0.41 (0.07)	0.38 (0.08)	0.39 (0.08)	0.38 (0.06)	0.38 (0.07)	0.34 (0.03)	0.34 (0.04)
MCD	0.18 (0.04)	0.17 (0.04)	0.18 (0.04)	0.17 (0.03)	0.17 (0.04)	0.14 (0.02)	0.14 (0.03)
GL	0.02 (0.03)	0.01 (0.01)	0.01 (0.02)	0.03 (0.02)	0.02 (0.02)	0.01 (0.03)	0.01 (0.02)
STAT	0.63 (0.05)	0.62 (0.05)	0.62 (0.05)	0.62 (0.04)	0.62 (0.04)	0.58 (0.04)	0.61 (0.04)
STRENGTH	0.51 (0.02)	0.47 (0.02)	0.47 (0.01)	0.46 (0.02)	0.47 (0.02)	0.44 (0.04)	0.47 (0.02)
RUMPWIDTH	0.56 (0.03)	0.53 (0.04)	0.53 (0.04)	0.52 (0.04)	0.52 (0.04)	0.49 (0.04)	0.52 (0.03)
Brown Swiss							
DCD	0.23 (0.12)	0.25 (0.12)	0.25 (0.12)	0.27 (0.11)	0.27 (0.11)	0.21 (0.15)	0.22 (0.15)
MCD	0.21 (0.03)	0.24 (0.05)	0.24 (0.05)	0.21 (0.08)	0.21 (0.08)	0.18 (0.13)	0.19 (0.13)
GL	0.08 (0.07)	0.14 (0.06)	0.14 (0.06)	0.16 (0.07)	0.16 (0.07)	0.14 (0.05)	0.15 (0.05)
STAT	0.44 (0.06)	0.41 (0.08)	0.41 (0.08)	0.41 (0.08)	0.41 (0.08)	0.36 (0.11)	0.38 (0.11)
STRENGTH	0.33 (0.07)	0.31 (0.03)	0.31 (0.03)	0.28 (0.07)	0.28 (0.07)	0.25 (0.04)	0.27 (0.05)
RUMPWIDTH	0.38 (0.06)	0.34 (0.04)	0.34 (0.04)	0.32 (0.02)	0.32 (0.02)	0.32 (0.03)	0.33 (0.03)

## Discussion

The genome-wide association analysis produced a description of regions of the genome affecting calving difficulty in Holstein, Brown Swiss and Jersey. The regions identified were in the proximity of previously described QTL that would most likely affect calving ease by altering the feto-pelvic proportion. Yet the network analysis pointed to a richer and more complex biology underlying dystocia in the different breeds. While most of the research regarding calving difficulty in dairy cattle has focused on the Holstein breed or its crosses, this paper provides and insight into calving difficulty in Brown Swiss and Jersey cattle. In our current analysis inclusion of network information did not increase accuracy of genomic prediction. This could be due to sample size and the use of mid density SNP arrays. Future investigations should focus on increasing the size of the populations and the density of the SNP available through the inclusion of female genotypes and imputed sequence information. While genotypes may be sufficient to predict performance in well-recorded and highly heritable traits, genomic selection on other traits may benefit from the incorporation of functional information and network structure into the predictions.

## Conclusions

In the current paper we have employed a gene network analysis to identify genomic regions associated with calving difficulty in dairy cattle. As expected, these analyses identified individual SNP associated with calving difficulty, and enriched pathways that included terms related to dystocia. Yet we did not identify a clear across breeds network and network inclusion did not increased predictive ability in genomic selection.

## Additional files

Additional file 1: **Figure S1.** Schematic representation of the workflow used in this study. (PDF 11 kb)

Additional file 2: **Figure S2.** Pearson correlation of de-regress PTA between the traits. Holstein, Brown Swiss and Jersey, respectively. (PDF 14 kb)

Additional file 3: **Figure S3.** Manhattan plots for PVg averaged across traits (Var). The green circles correspond to SNP that were declared significant in all the traits evaluated. (PDF 305 kb)

Additional file 4: **Figure S4.** Network diagrams for HO, BS and JE respectively. Green dots correspond to genes enriching significant GO terms. Red dots correspond to genes enriching significant GO terms and located within dystocia QTLs. Blue dots correspond to the remaining of the genes highly correlated among them (r_xy|z_ ≥ 0.98). Yellow circles correspond to the most connected genes within dystocia QTL (see Table 3). (PDF 819 kb)

Additional file 5: **Figure S5.** Single-SNP Manhattan plots for the percentage of genetic variance adsorbed for each trait in the three breeds. (PNG 249 kb)

Additional file 6: **Figure S6.** Single-SNP Manhattan plots for the percentage of genetic variance adsorbed for each trait in the three breeds. (PNG 311 kb)

Additional file 7: **Figure S7.** Single-SNP Manhattan plots for the percentage of genetic variance adsorbed for each trait in the three breeds. (PNG 224 kb)

Additional file 8: **Figure S8.** Single-SNP Manhattan plots for the percentage of genetic variance adsorbed for each trait in the three breeds. (PNG 281 kb)

Additional file 9: **Figure S9.** Single-SNP Manhattan plots for the percentage of genetic variance adsorbed for each trait in the three breeds. (PNG 231 kb)

Additional file 10: **Figure S10.** Single-SNP Manhattan plots for the percentage of genetic variance adsorbed for each trait in the three breeds. (PNG 374 kb)

Additional file 11: **Table S1.** Proportion of variance absorbed by different genomic relationship matrices in the HO population. (DOCX 22 kb)

Additional file 12: **Table S2.** Proportion of variance absorbed by different genomic relationship matrices in the JE population. (DOCX 22 kb)

Additional file 13: **Table S3.** Proportion of variance absorbed by different genomic relationship matrices in the BS population. (DOCX 22 kb)

## Abbreviations

AGIL: Animal Genomics and Improvement Laboratory
AWM: Association weight matrix
AYB: Average year of birth
bp: Base pair
BS: Brown Swiss
DCD: Sire calving difficulty
FDR: False discovery rate
GL: Gestation length
GO: Gene ontology
GWAS: Genome-wide association studies
HO: Holstein
JE: Jersey
KEGG: Kyoto encyclopedia of genes and genomes
LD: Linkage disequilibrium
Mbp: Mega base pair
MCD: Maternal grand sire calving difficulty
MGS: Maternal grand sire
PA: Parent average
PCIT: Partial correlation information theory
PTA: Predicted transmitted ability
PV: Proportion of genetic variance
QTL: Quantitative trait loci
RW: Rump width
SNP: Single nucleotide polymorphisms
STAT: Stature
STR: Strength
