# Supplementary figures and images for: Including gene networks to predict calving difficulty in Holstein, Brown Swiss and Jersey cattle
Source: BMC Genet. 2018 Apr 2;19:20. doi: 10.1186/s12863-018-0606-y (PMC5880070; doi:10.1186/s12863-018-0606-y)

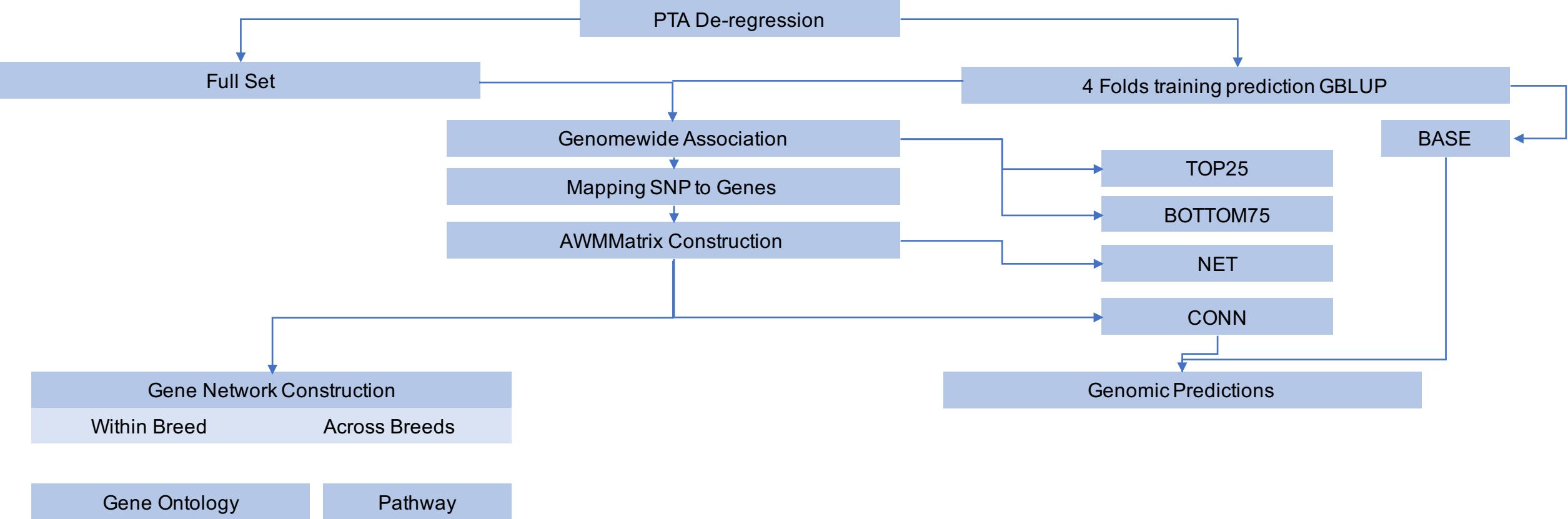

BASE= All SNP

TOP25= Top 25 GWAS SNP

BOTTOM75= BOTTOM75 GWAS SNP

NET= AWM SNP

CONN= AWM SNP with connections

Supplement: Supplementary file 1 — Figure S1. Schematic representation of the workflow used in this study. (PDF 11 kb) [file 12863_2018_606_MOESM1_ESM.pdf]

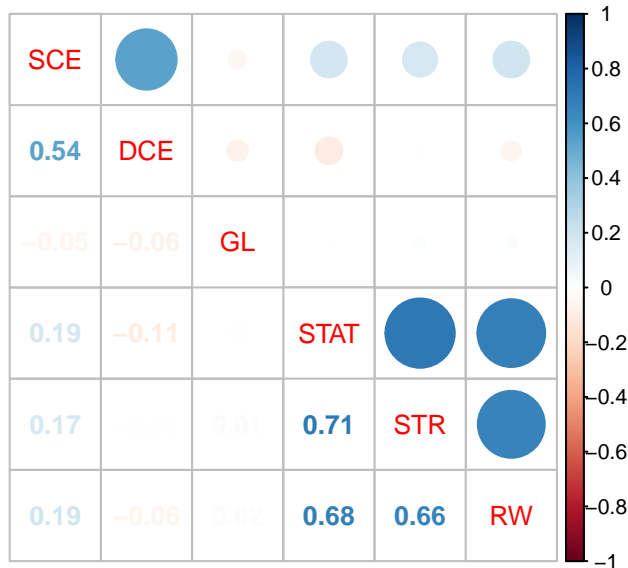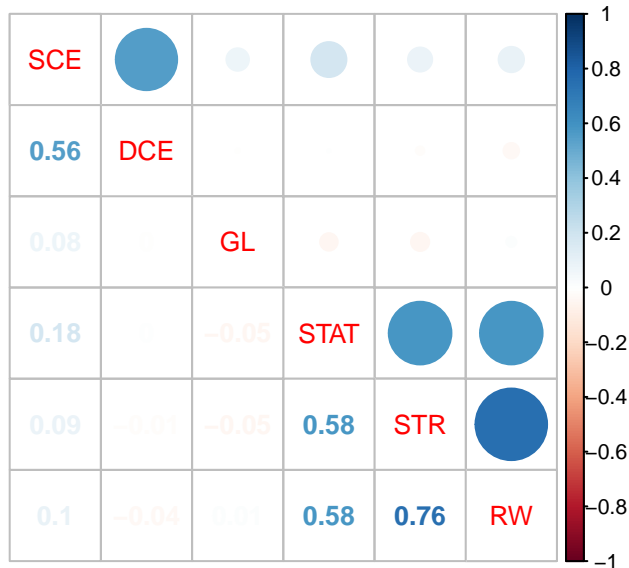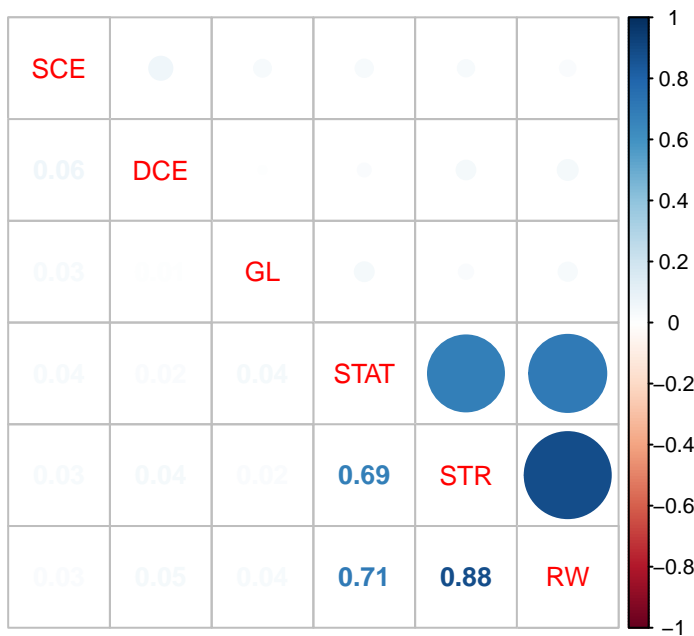

Supplement: Supplementary file 2 — Figure S2. Pearson correlation of de-regress PTA between the traits. Holstein, Brown Swiss and Jersey, respectively. (PDF 14 kb) [file 12863_2018_606_MOESM2_ESM.pdf]

HO

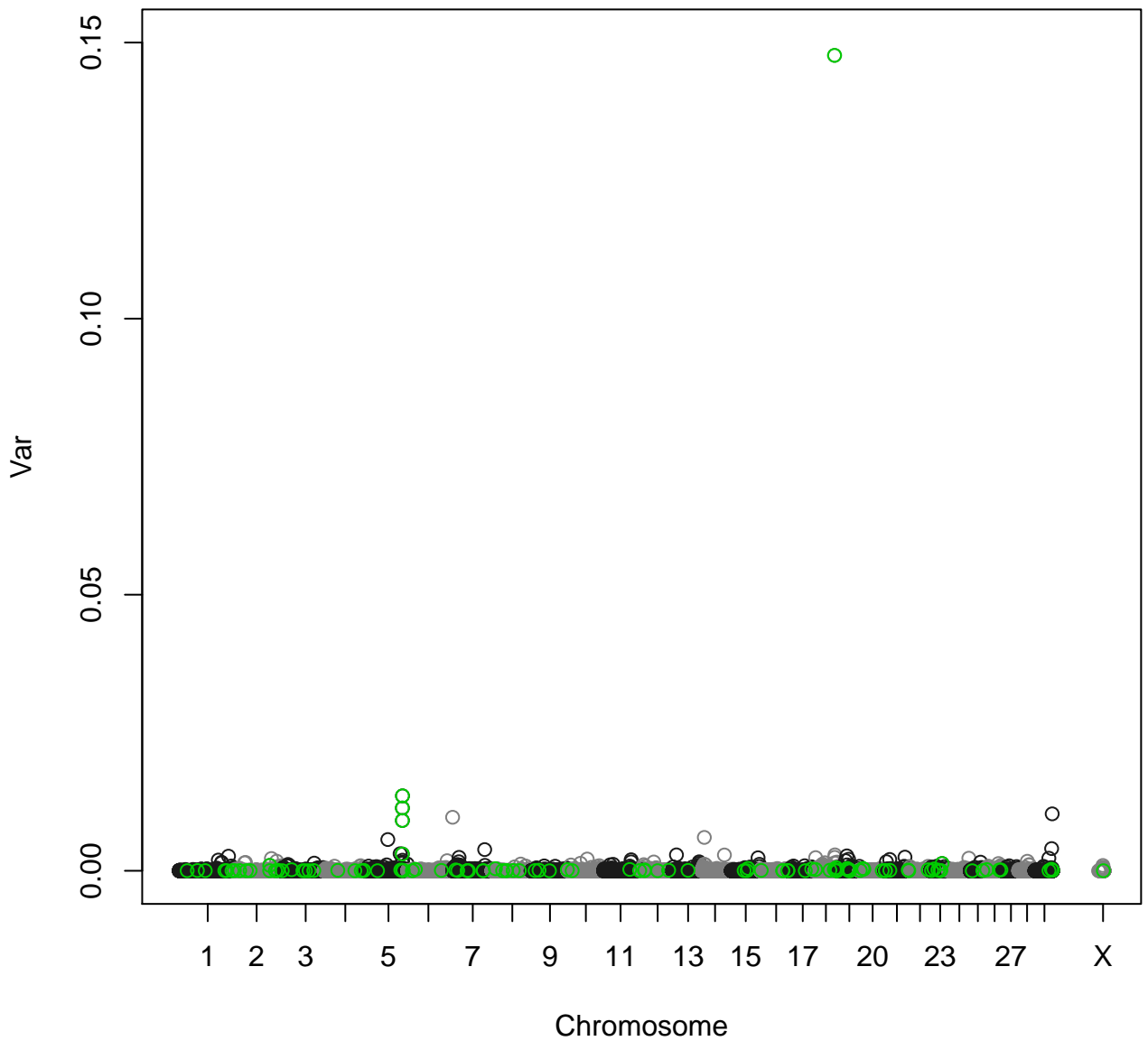

BS

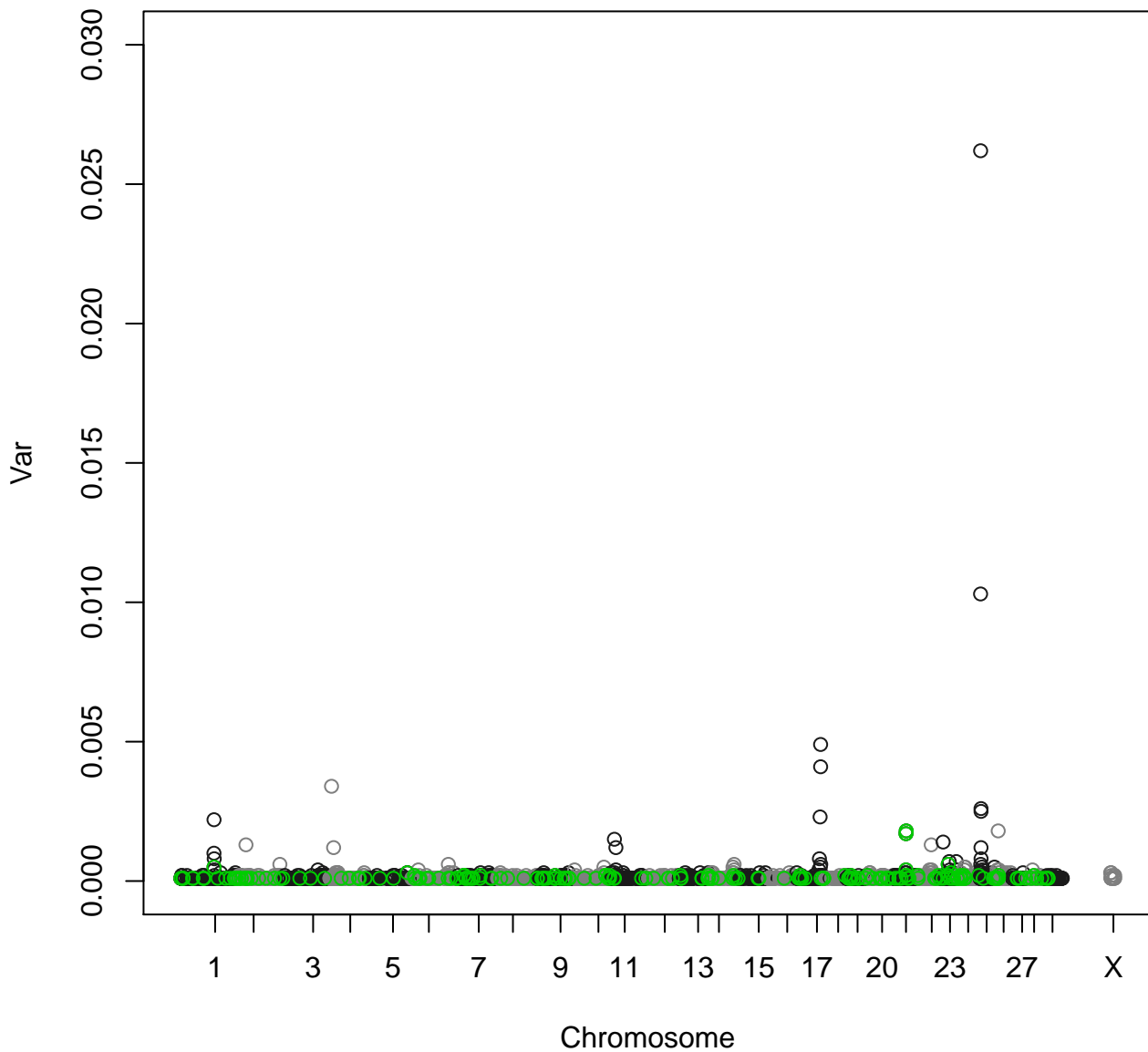

JE

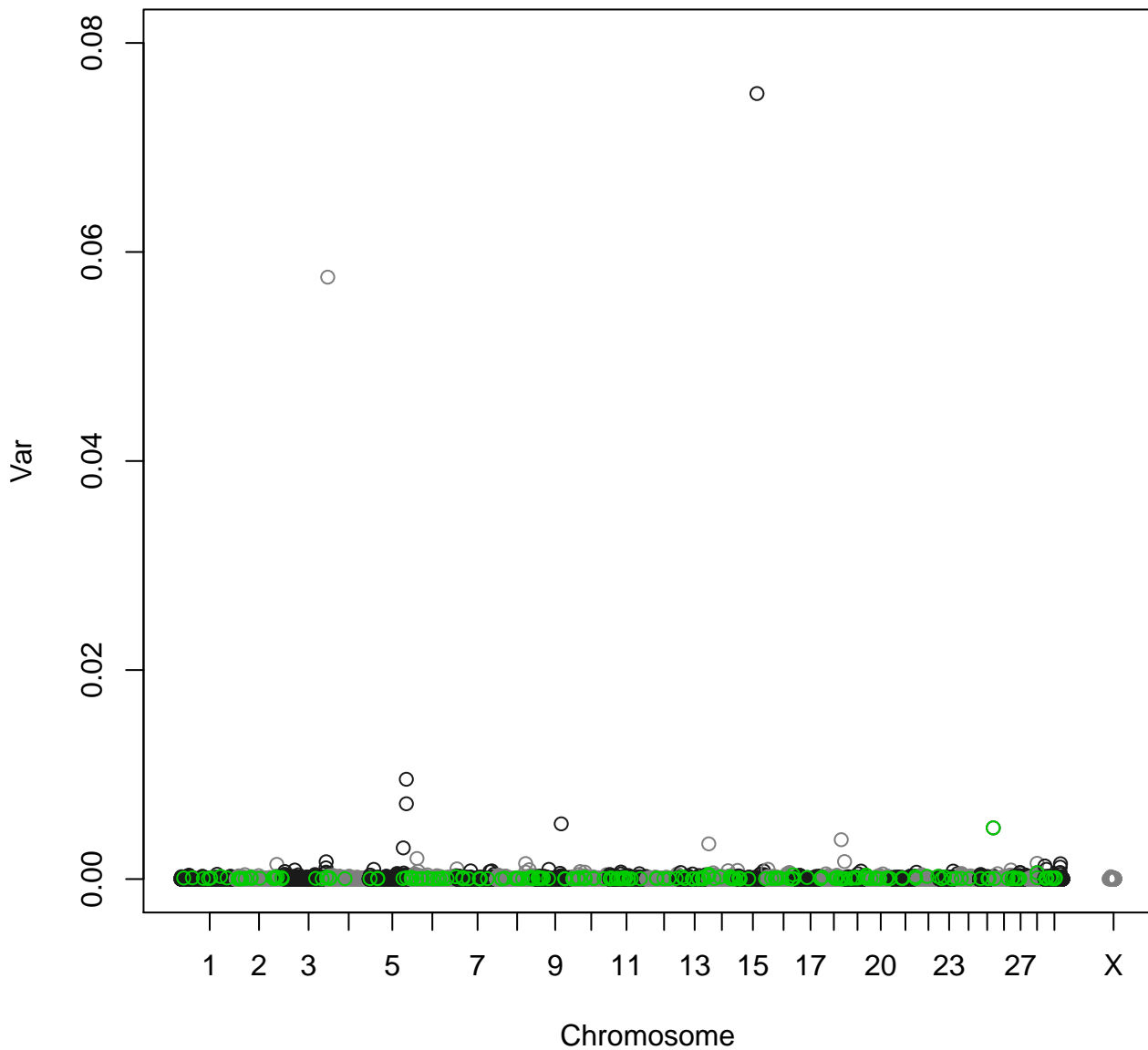

Supplement: Supplementary file 3 — Figure S3. Manhattan plots for PVg averaged across traits (Var). The green circles correspond to SNP that were declared significant in all the traits evaluated. (PDF 305 kb) [file 12863_2018_606_MOESM3_ESM.pdf]

HO

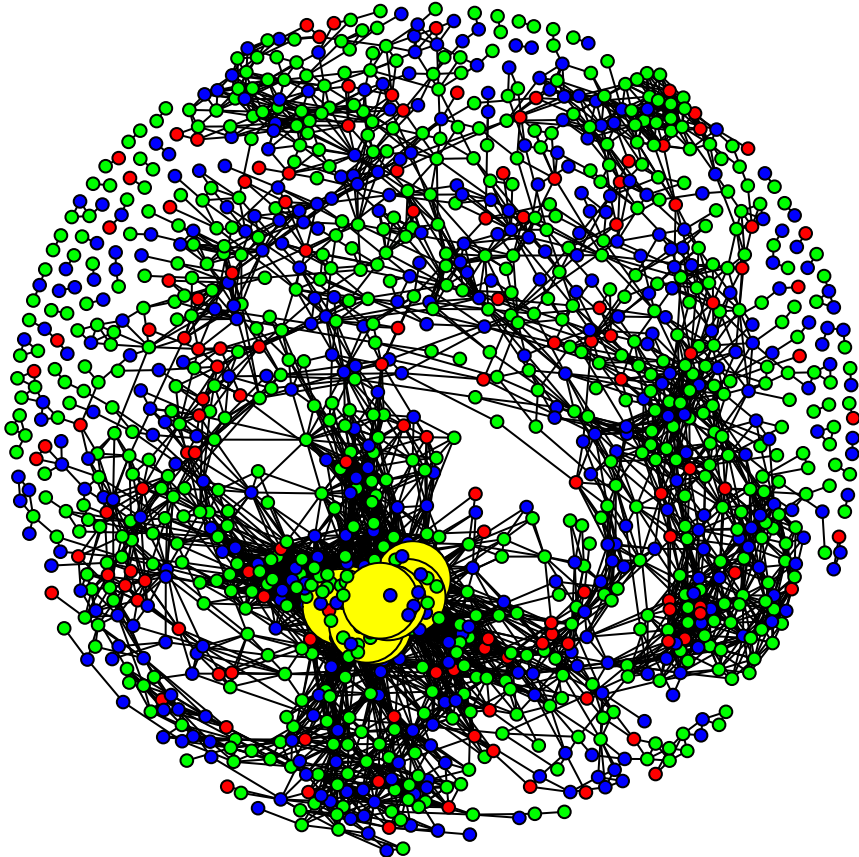

**BS**

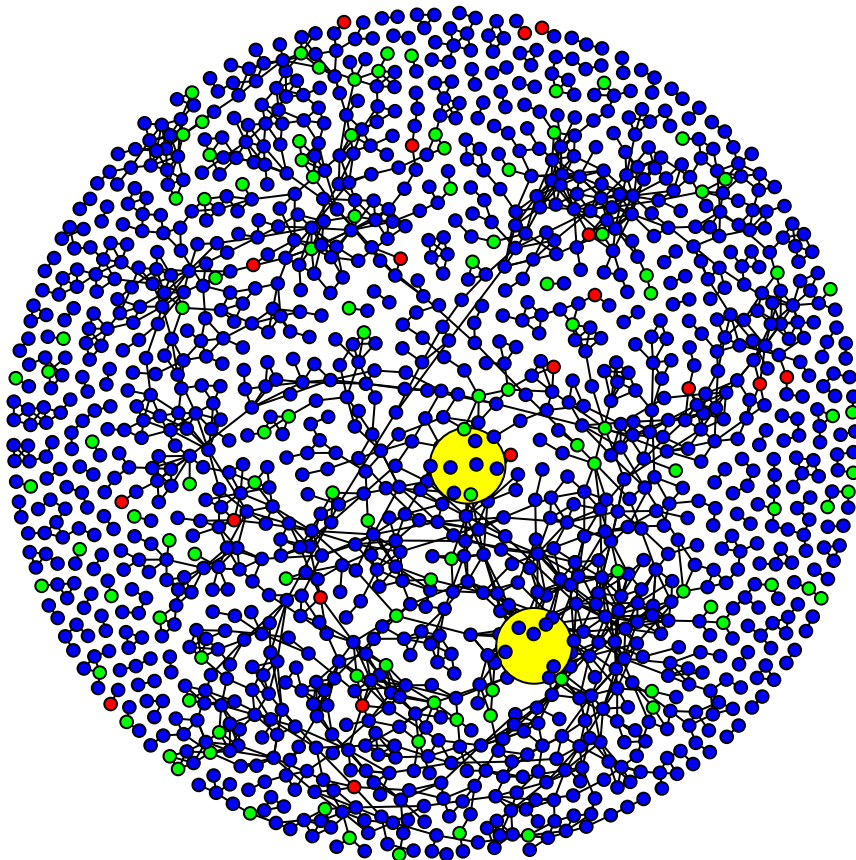

JE

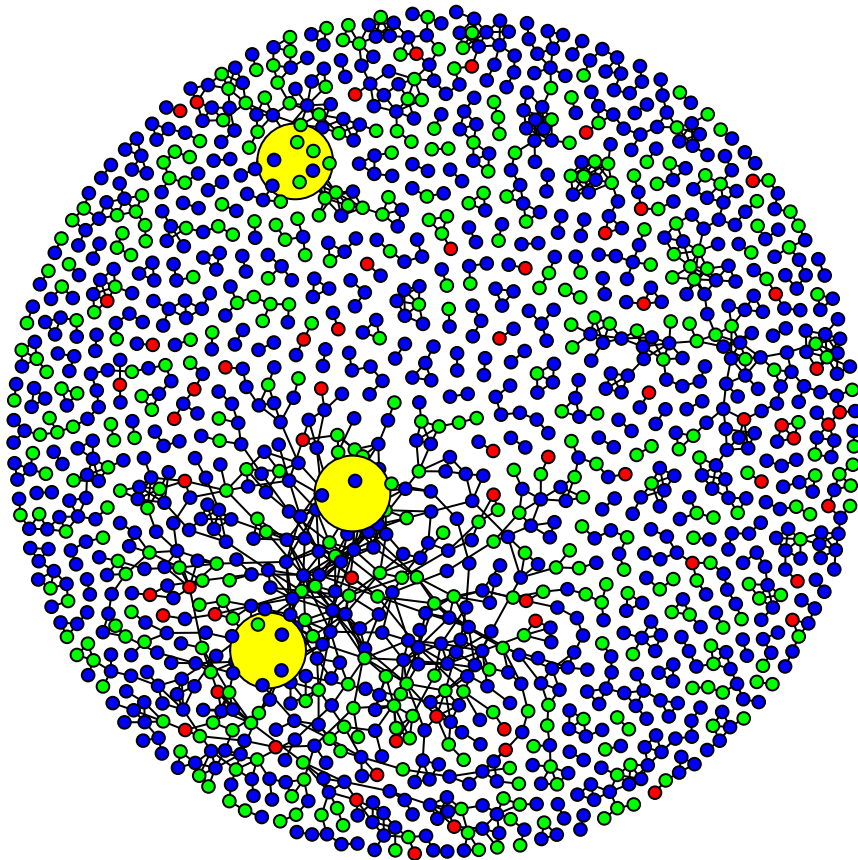

Supplement: Supplementary file 4 — Figure S4. Network diagrams for HO, BS and JE respectively. Green dots correspond to genes enriching significant GO terms. Red dots correspond to genes enriching significant GO terms and located within dystocia QTLs. Blue dots correspond to the remaining of the genes highly correlated among them (rxy|z ≥ 0.98). Yellow circles correspond to the most connected genes within dystocia QTL (see Table 3). (PDF 819 kb) [file 12863_2018_606_MOESM4_ESM.pdf]

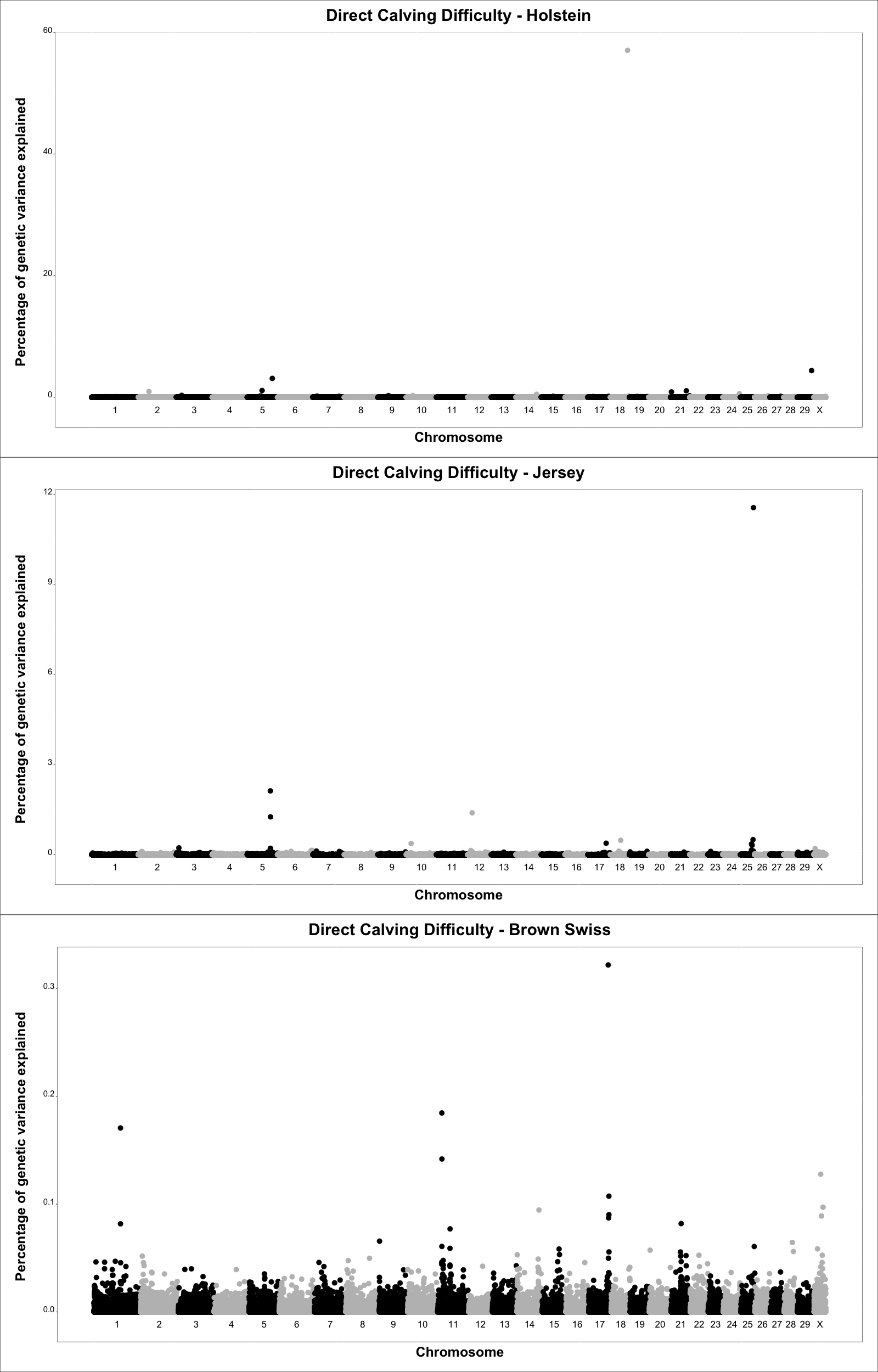

Supplement: Supplementary file 5 — Figure S5. Single-SNP Manhattan plots for the percentage of genetic variance adsorbed for each trait in the three breeds. (PNG 249 kb) [file 12863_2018_606_MOESM5_ESM.png]

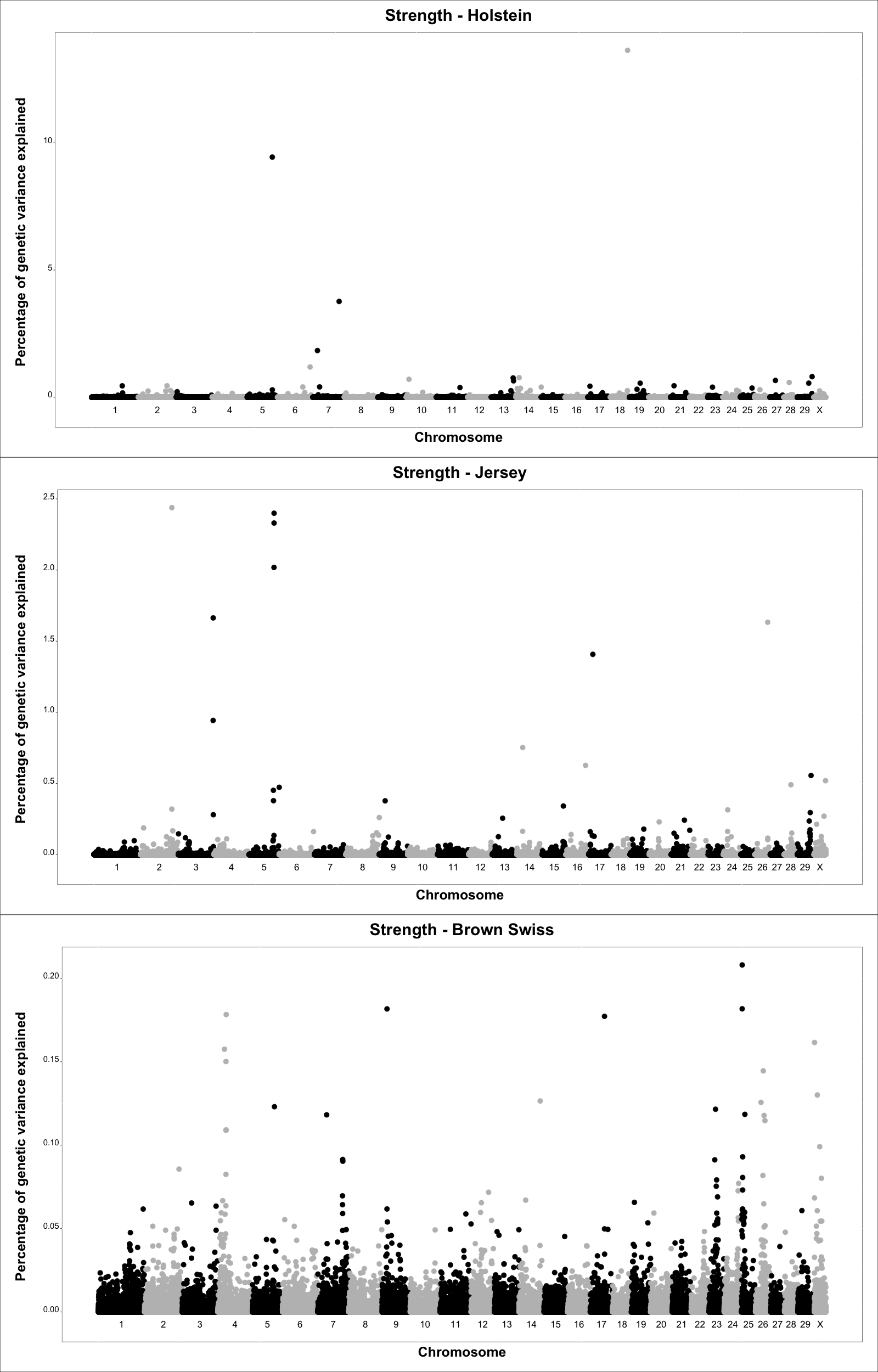

Supplement: Supplementary file 6 — Figure S6. Single-SNP Manhattan plots for the percentage of genetic variance adsorbed for each trait in the three breeds. (PNG 311 kb) [file 12863_2018_606_MOESM6_ESM.png]

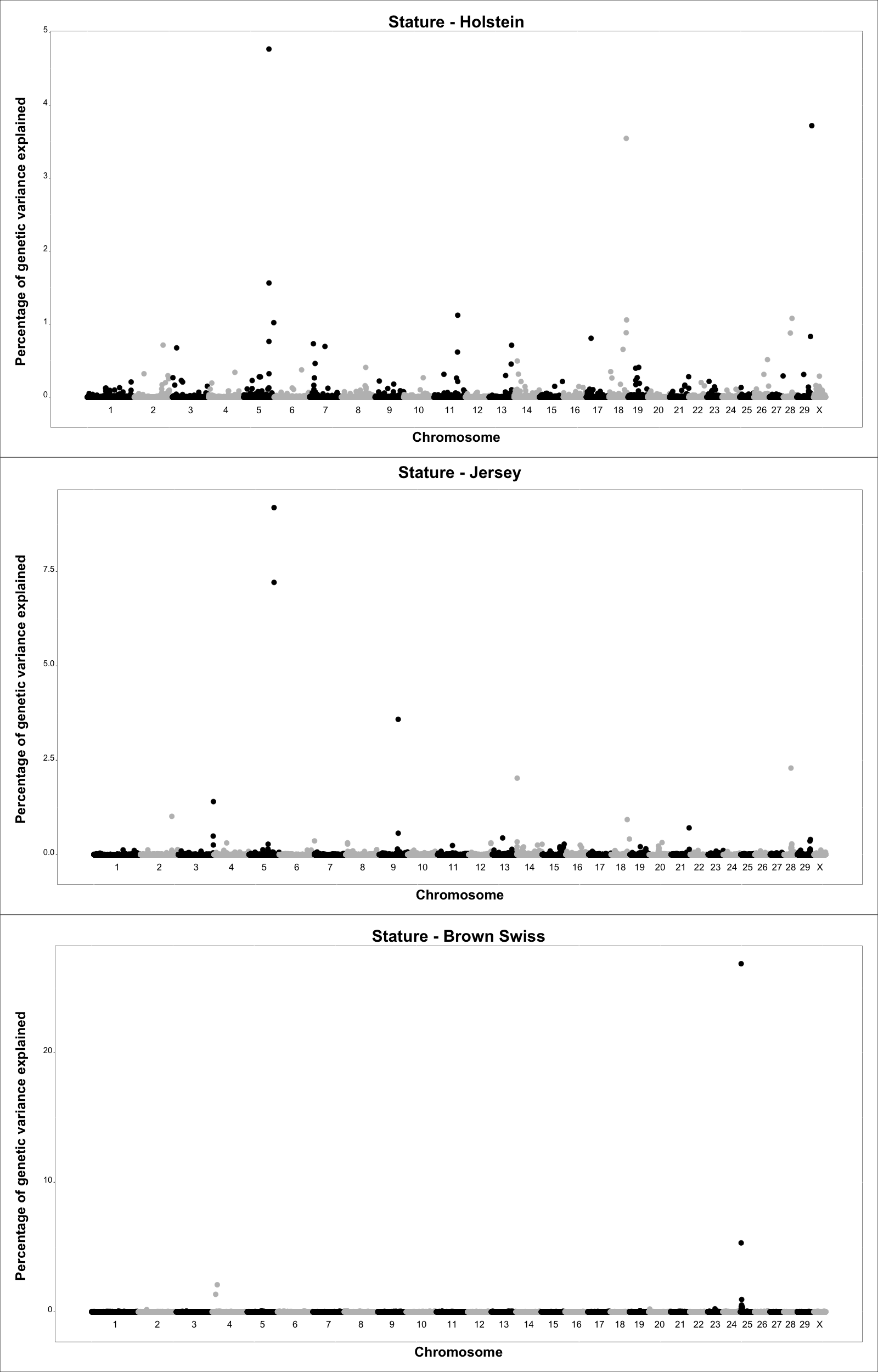

Supplement: Supplementary file 7 — Figure S7. Single-SNP Manhattan plots for the percentage of genetic variance adsorbed for each trait in the three breeds. (PNG 224 kb) [file 12863_2018_606_MOESM7_ESM.png]

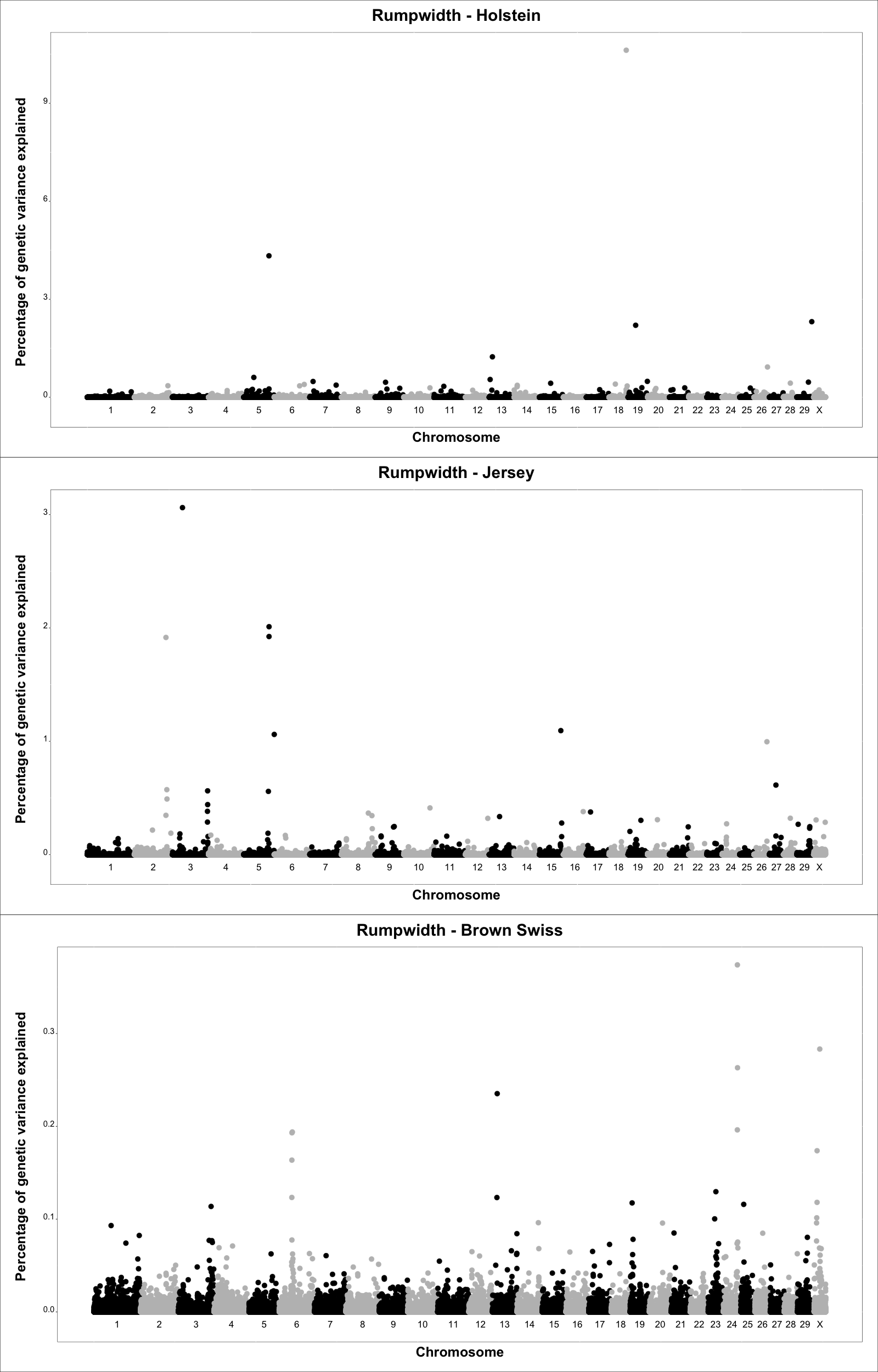

Supplement: Supplementary file 8 — Figure S8. Single-SNP Manhattan plots for the percentage of genetic variance adsorbed for each trait in the three breeds. (PNG 281 kb) [file 12863_2018_606_MOESM8_ESM.png]

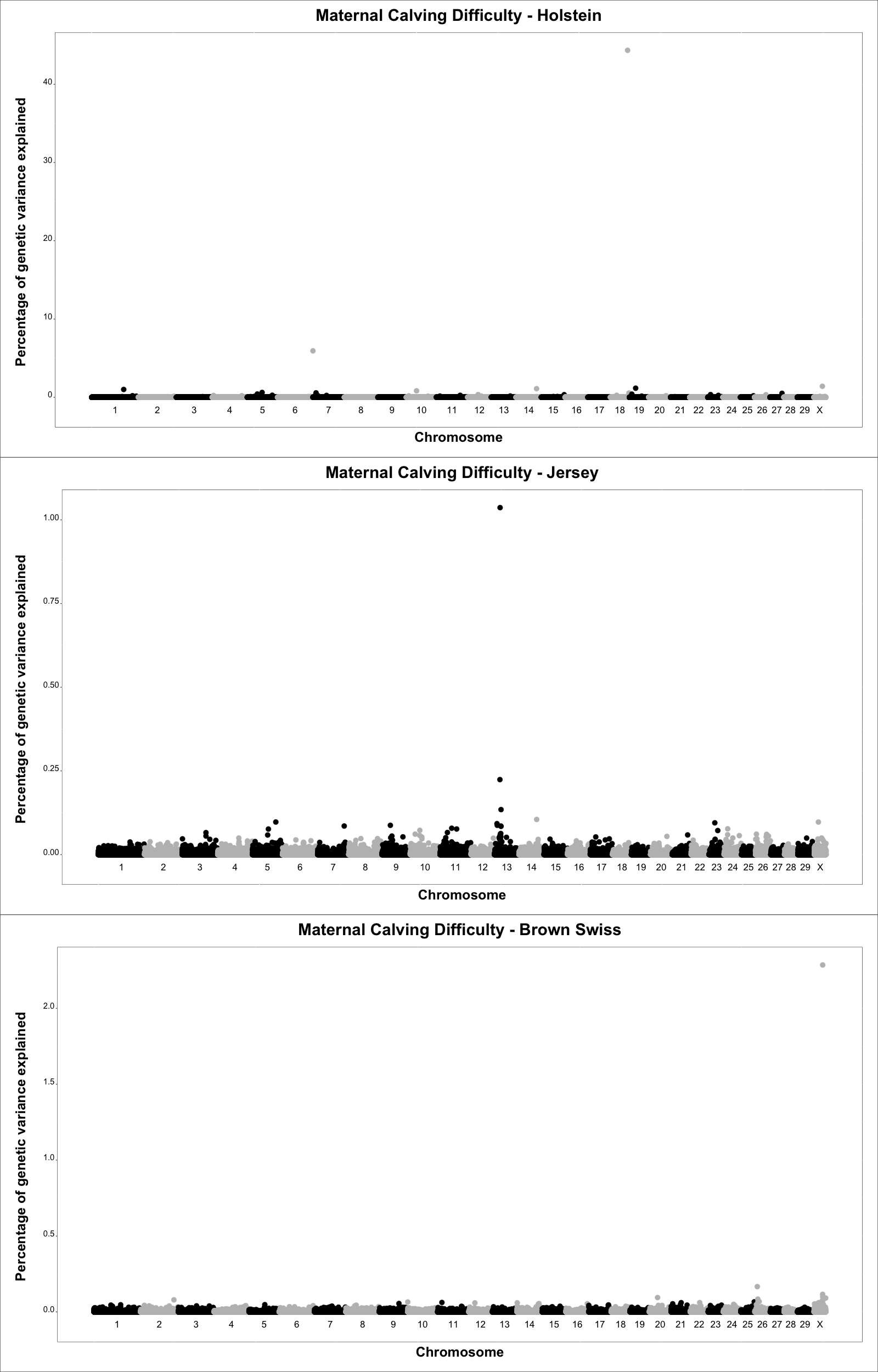

Supplement: Supplementary file 9 — Figure S9. Single-SNP Manhattan plots for the percentage of genetic variance adsorbed for each trait in the three breeds. (PNG 231 kb) [file 12863_2018_606_MOESM9_ESM.png]

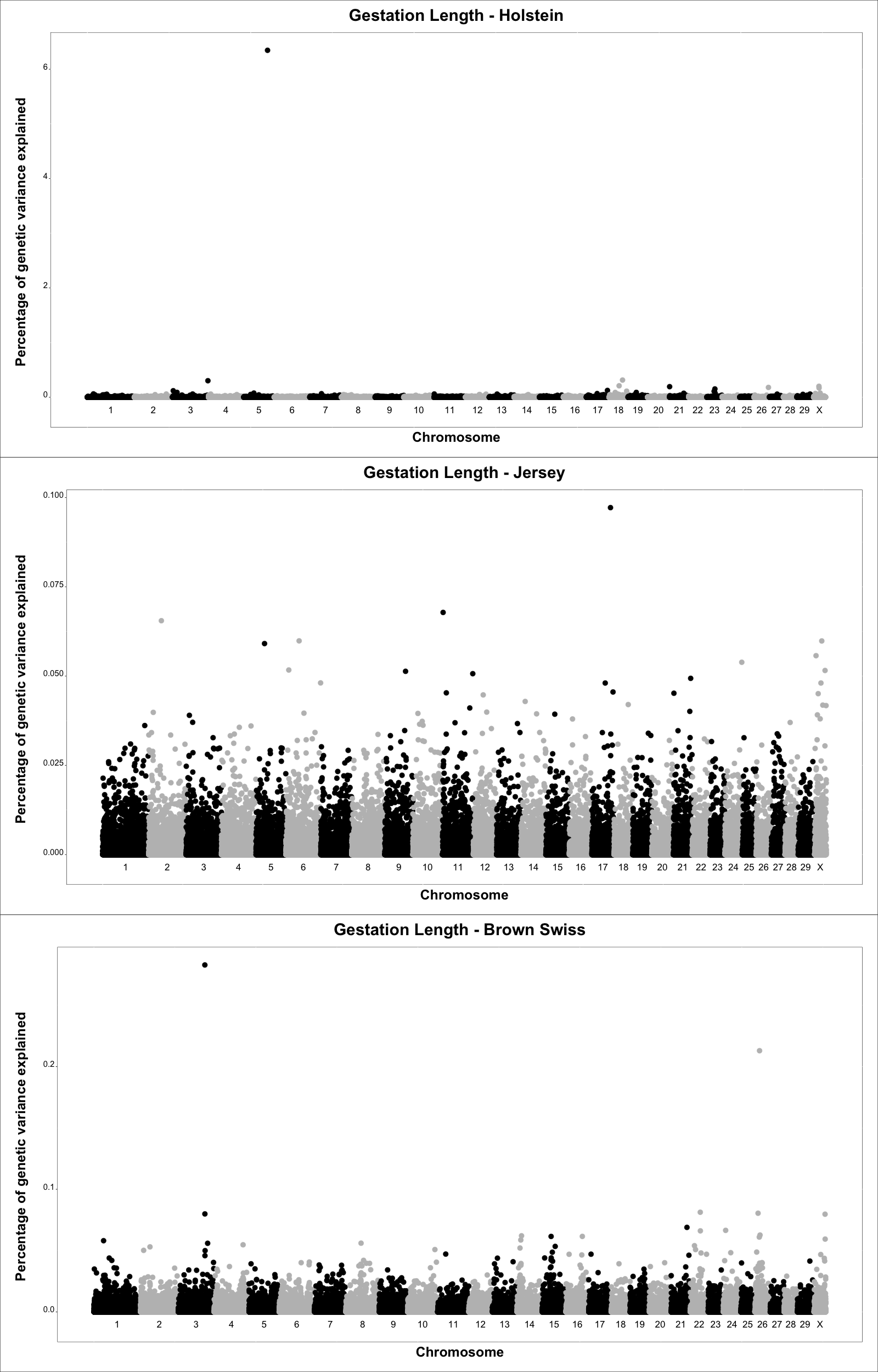

Supplement: Supplementary file 10 — Figure S10. Single-SNP Manhattan plots for the percentage of genetic variance adsorbed for each trait in the three breeds. (PNG 374 kb) [file 12863_2018_606_MOESM10_ESM.png]
