# Supplementary material for: Including gene networks to predict calving difficulty in Holstein, Brown Swiss and Jersey cattle
Source: BMC Genet. 2018 Apr 2;19:20. doi: 10.1186/s12863-018-0606-y (PMC5880070; doi:10.1186/s12863-018-0606-y)
Supplement: Supplementary file 11 — Table S1. Proportion of variance absorbed by different genomic relationship matrices in the HO population. (DOCX 22 kb) [file 12863_2018_606_MOESM11_ESM.docx]

**Table S1.** Proportion of variance absorbed by different genomic relationship matrices in the Holstein population.

| Trait/Model | BASE | TOP25 | BOT75 | NET | CONN | FREE |
| --- | --- | --- | --- | --- | --- | --- |
| DCD |  |  |  |  |  |  |
| 1 | 0.39 (0.011) | . | . | . | . | . |
| 2 | . | 0.42 (0.014) | . | . | . | . |
| 3 | . | 0.41 (0.014) | 0.01 (0.002) | . | . | . |
| 4 | . | . | . | 0.35 (0.015) | . | . |
| 5 | . | . | . | 0.29 (0.016) | . | 0.13 (0.014) |
| 6 | . | . | . | . | 0.29 (0.043) | . |
| 7 | . | . | . | . | 0.23 (0.04) | 0.17 (0.016) |
| MCD |  |  |  |  |  |  |
| 1 | 0.43 (0.009) | . | . | . | . | . |
| 2 | . | 0.43 (0.019) | . | . | . | . |
| 3 | . | 0.42 (0.024) | 0.01 (0.009) | . | . | . |
| 4 | . | . | . | 0.36 (0.019) | . | . |
| 5 | . | . | . | 0.26 (0.022) | . | 0.19 (0.016) |
| 6 | . | . | . | . | 0.31 (0.042) | . |
| 7 | . | . | . | . | 0.2 (0.032) | 0.23 (0.015) |
| GL |  |  |  |  |  |  |
| 1 | 0.14 (0.032) | . | . | . | . | . |
| 2 | . | 0.15 (0.03) | . | . | . | . |
| 3 | . | 0.06 (0.011) | 0.1 (0.019) | . | . | . |
| 4 | . | . | . | 0.12 (0.029) | . | . |
| 5 | . | . | . | 0.08 (0.024) | . | 0.08 (0.017) |
| 6 | . | . | . | . | 0.09 (0.023) | . |
| 7 | . | . | . | . | 0.06 (0.014) | 0.07 (0.016) |
| STAT |  |  |  |  |  |  |
| 1 | 0.74 (0.006) | . | . | . | . | . |
| 2 | . | 0.67 (0.01) | . | . | . | . |
| 3 | . | 0.47 (0.136) | 0.21 (0.139) | . | . | . |
| 4 | . | . | . | 0.56 (0.015) | . | . |
| 5 | . | . | . | 0.38 (0.01) | . | 0.36 (0.016) |
| 6 | . | . | . | . | 0.45 (0.05) | . |
| 7 | . | . | . | . | 0.23 (0.035) | 0.46 (0.025) |
| STRE |  |  |  |  |  |  |
| 1 | 0.66 (0.01) | . | . | . | . | . |
| 2 | . | 0.61 (0.006) | . | . | . | . |
| 3 | . | 0.5 (0.107) | 0.13 (0.104) | . | . | . |
| 4 | . | . | . | 0.52 (0.016) | . | . |
| 5 | . | . | . | 0.39 (0.014) | . | 0.29 (0.014) |
| 6 | . | . | . | . | 0.38 (0.034) | . |
| 7 | . | . | . | . | 0.24 (0.032) | 0.37 (0.027) |
| RUMP |  |  |  |  |  |  |
| 1 | 0.69 (0.004) | . | . | . | . | . |
| 2 | . | 0.63 (0.005) | . | . | . | . |
| 3 | . | 0.58 (0.052) | 0.07 (0.052) | . | . | . |
| 4 | . | . | . | 0.53 (0.019) | . | . |
| 5 | . | . | . | 0.37 (0.024) | . | 0.33 (0.029) |
| 6 | . | . | . | . | 0.41 (0.072) | . |
| 7 | . | . | . | . | 0.41 (0.041) | 0.23 (0.048) |
