# Supplementary material for: Including gene networks to predict calving difficulty in Holstein, Brown Swiss and Jersey cattle
Source: BMC Genet. 2018 Apr 2;19:20. doi: 10.1186/s12863-018-0606-y (PMC5880070; doi:10.1186/s12863-018-0606-y)
Supplement: Supplementary file 12 — Table S2. Proportion of variance absorbed by different genomic relationship matrices in the JE population. (DOCX 22 kb) [file 12863_2018_606_MOESM12_ESM.docx]

**Table S2.** Proportion of variance absorbed by different genomic relationship matrices in the Jersey population.

| Trait/Model | BASE | TOP25 | BOT75 | NET | CONN | FREE |
| --- | --- | --- | --- | --- | --- | --- |
| DCD |  |  |  |  |  |  |
| 1 | 0.43 (0.036) | . | . | . | . | . |
| 2 | . | 0.45 (0.034) | . | . | . | . |
| 3 | . | 0.23 (0.016) | 0.23 (0.025) | . | . | . |
| 4 | . | . | . | 0.48 (0.038) | . | . |
| 5 | . | . | . | 0.41 (0.075) | . | 0.06 (0.04) |
| 6 | . | . | . | . | 0.6 (0.073) | . |
| 7 | . | . | . | . | 0.59 (0.077) | 0.02 (0.01) |
| MCD |  |  |  |  |  |  |
| 1 | 0.26 (0.024) | . | . | . | . | . |
| 2 | . | 0.32 (0.016) |  | . | . | . |
| 3 | . | 0.31 (0.01) | 0.02 (0.022) | . | . | . |
| 4 | . | . | . | 0.28 (0.022) | . | . |
| 5 | . | . | . | 0.17 (0.119) | . | 0.1 (0.114) |
| 6 | . | . | . | . | 0.61 (0.059) | . |
| 7 | . | . | . | . | 0.6 (0.06) | 0.01 (0.002) |
| GL |  |  |  |  |  |  |
| 1 | 0.06 (0.005) | . | . | . | . | . |
| 2 | . | 0.09 (0.021) | . | . | . | . |
| 3 | . | 0.06 (0.011) | 0.06 (0.008) | . | . | . |
| 4 | . | . | . | 0.07 (0.007) | . | . |
| 5 | . | . | . | 0.05 (0.006) | . | 0.05 (0.004) |
| 6 | . | . | . | . | 0.48 (0.083) | . |
| 7 | . | . | . | . | 0.48 (0.079) | 0.03 (0.005) |
| STAT |  |  |  |  |  |  |
| 1 | 0.74 (0.032) | . | . | . | . | . |
| 2 | . | 0.83 (0.024) | . | . | . | . |
| 3 | . | 0.81 (0.023) | 0.02 (0.002) | . | . | . |
| 4 | . | . | . | 0.79 (0.019) | . | . |
| 5 | . | . | . | 0.79 (0.019) | . | 0.01 (0.001) |
| 6 | . | . | . | . | 0.78 (0.046) | . |
| 7 | . | . | . | . | 0.74 (0.054) | 0.1 (0.03) |
| STRE |  |  |  |  |  |  |
| 1 | 0.66 (0.027) | . | . | . | . | . |
| 2 | . | 0.81 (0.022) | . | . | . | . |
| 3 | . | 0.6 (0.367) | 0.21 (0.358) | . | . | . |
| 4 | . | . | . | 0.77 (0.022) | . | . |
| 5 | . | . | . | 0.76 (0.023) | . | 0.01 (0.002) |
| 6 | . | . | . | . | 0.73 (0.056) | . |
| 7 | . | . | . | . | 0.7 (0.065) | 0.1 (0.038) |
| RUMP |  |  |  |  |  |  |
| 1 | 0.69 (0.017) | . | . | . | . | . |
| 2 | . | 0.82 (0.016) |  | . | . | . |
| 3 | . | 0.53 (0.348) | 0.3 (0.357) | . | . | . |
| 4 | . | . | . | 0.79 (0.01) | . | . |
| 5 | . | . | . | 0.78 (0.01) | . | 0.01 (0.002) |
| 6 | . | . | . | . | 0.75 (0.05) | . |
| 7 | . | . | . | . | 0.73 (0.057) | 0.09 (0.034) |
