# Supplementary material for: Including gene networks to predict calving difficulty in Holstein, Brown Swiss and Jersey cattle
Source: BMC Genet. 2018 Apr 2;19:20. doi: 10.1186/s12863-018-0606-y (PMC5880070; doi:10.1186/s12863-018-0606-y)
Supplement: Supplementary file 13 — Table S3. Proportion of variance absorbed by different genomic relationship matrices in the BS population. (DOCX 22 kb) [file 12863_2018_606_MOESM13_ESM.docx]

**Table S3.** Proportion of variance absorbed by different genomic relationship matrices in the Brown Swiss population.

| Trait/Model | BASE | TOP25 | BOT75 | NET | CONN | FREE |
| --- | --- | --- | --- | --- | --- | --- |
| DCD |  |  |  |  |  |  |
| 1 | 0.24 (0.1) | . | . | . | . | . |
| 2 | . | 0.62 (0.062) | . | . | . | . |
| 3 | . | 0.42 (0.278) | 0.2 (0.337) | . | . | . |
| 4 | . | . | . | 0.55 (0.066) | . | . |
| 5 | . | . | . | 0.55 (0.066) | . | 0.01 (0.001) |
| 6 | . | . | . | . | 0.79 (0.085) | . |
| 7 | . | . | . | . | 0.8 (0.074) | 0.02 (0.019) |
| MCD |  |  |  |  |  |  |
| 1 | 0.08 (0.053) | . | . | . | . | . |
| 2 | . | 0.61 (0.106) | . | . | . | . |
| 3 | . | 0.36 (0.321) | 0.24 (0.304) | . | . | . |
| 4 | . | . | . | 0.52 (0.104) | . | . |
| 5 | . | . | . | 0.51 (0.108) | . | 0.01 (0.001) |
| 6 | . | . | . | . | 0.8 (0.054) | . |
| 7 | . | . | . | . | 0.8 (0.053) | 0.01 (0.012) |
| GL |  |  |  |  |  |  |
| 1 | 0.05 (0.013) | . | . | . | . | . |
| 2 | . | 0.19 (0.201) | . | . | . | . |
| 3 | . | 0.13 (0.135) | 0.09 (0.042) | . | . | . |
| 4 | . | . | . | 0.16 (0.092) | . | . |
| 5 | . | . | . | 0.15 (0.086) | . | 0.04 (0.005) |
| 6 | . | . | . | . | 0.82 (0.016) | . |
| 7 | . | . | . | . | 0.82 (0.019) | 0.01 (0.004) |
| STAT |  |  |  |  |  |  |
| 1 | 0.74 (0.025) | . | . | . | . | . |
| 2 | . | 0.84 (0.008) | . | . | . | . |
| 3 | . | 0.75 (0.168) | 0.09 (0.161) | . | . | . |
| 4 | . | . | . | 0.81 (0.009) | . | . |
| 5 | . | . | . | 0.81 (0.01) | . | 0.01 (0.001) |
| 6 | . | . | . | . | 0.87 (0.064) | . |
| 7 | . | . | . | . | 0.87 (0.065) | 0.03 (0.021) |
| STRE |  |  |  |  |  |  |
| 1 | 0.57 (0.072) | . | . | . | . | . |
| 2 | . | 0.81 (0.023) | . | . | . | . |
| 3 | . | 0.78 (0.019) | 0.03 (0.005) | . | . | . |
| 4 | . | . | . | 0.8 (0.044) | . | . |
| 5 | . | . | . | 0.79 (0.042) | . | 0.02 (0) |
| 6 | . | . | . | . | 0.87 (0.018) | . |
| 7 | . | . | . | . | 0.86 (0.023) | 0.03 (0.015) |
| RUMP |  |  |  |  |  |  |
| 1 | 0.64 (0.019) | . | . | . | . | . |
| 2 | . | 0.82 (0.011) | . | . | . | . |
| 3 | . | 0.76 (0.018) | 0.06 (0.013) | . | . | . |
| 4 | . | . | . | 0.8 (0.027) | . | . |
| 5 | . | . | . | 0.78 (0.026) | . | 0.02 (0.001) |
| 6 | . | . | . | . | 0.85 (0.033) | . |
| 7 | . | . | . | . | 0.85 (0.036) | 0.02 (0.007) |
